# Targeting DNM1L/DRP1-FIS1 axis inhibits high-grade glioma progression by impeding mitochondrial respiratory cristae remodeling

**DOI:** 10.1186/s13046-024-03194-6

**Published:** 2024-09-30

**Authors:** Xiaodong Li, Jingjing Tie, Yuze Sun, Chengrong Gong, Shizhou Deng, Xiyu Chen, Shujiao Li, Yaoliang Wang, Zhenhua Wang, Feifei Wu, Hui Liu, Yousheng Wu, Guopeng Zhang, Qingdong Guo, Yanling Yang, Yayun Wang

**Affiliations:** 1 Specific Lab for Mitochondrial Plasticity Underlying Nervous System Diseases, National Demonstration Center for Experimental Preclinical Medicine Education, The Fourth Military Medical University, Xi’an, 710032 China; 2 Department of Hepatobiliary Surgery, Xi-Jing Hospital, The Fourth Military Medical University, Xi’an, 710032 China; 3 Department of Neurosurgery, Xi-Jing Hospital, The Fourth Military Medical University, Xi’an, 710032 China; 4 Department of Computer Fundamentals, The Fourth Military Medical University, Xi’an, 710032 China; 5 Department of Human Anatomy, Histology and Embryology, Medical School of Yan’an University, Yan’an, China

**Keywords:** Glioma, Mitochondrial respiratory cristae, OXPHOS, DNM1L/DRP1, FIS1, Mitophagy

## Abstract

**Background:**

The dynamics of mitochondrial respiratory cristae (MRC) and its impact on oxidative phosphorylation (OXPHOS) play a crucial role in driving the progression of high-grade glioma (HGG). However, the underlying mechanism remains unclear.

**Methods:**

In the present study, we employed machine learning-based transmission electron microscopy analysis of 7141 mitochondria from 54 resected glioma patients. Additionally, we conducted bioinformatics analysis and multiplex immunohistochemical (mIHC) staining of clinical glioma microarrays to identify key molecules involved in glioma. Subsequently, we modulated the expression levels of mitochondrial dynamic-1-like protein (DNM1L/DRP1), and its two receptors, mitochondrial fission protein 1 (FIS1) and mitochondrial fission factor (MFF), via lentiviral transfection to further investigate the central role of these molecules in the dynamics of glioblastoma (GBM) cells and glioma stem cells (GSCs). We then evaluated the potential impact of DNM1L/DRP1, FIS1, and MFF on the proliferation and progression of GBM cells and GSCs using a combination of CCK-8 assay, Transwell assay, Wound Healing assay, tumor spheroid formation assay and cell derived xenograft assay employing NOD/ShiLtJGpt-*Prkdc*^em26Cd52^*Il2rg*^em26Cd22^/Gpt (NCG) mouse model. Subsequently, we validated the ability of the DNM1L/DRP1-FIS1 axis to remodel MRC structure through mitophagy by utilizing Seahorse XF analysis technology, mitochondrial function detection, MRC abundance detection and monitoring dynamic changes in mitophagy.

**Results:**

Our findings revealed that compared to low-grade glioma (LGG), HGG exhibited more integrated MRC structures. Further research revealed that DNM1L/DRP1, FIS1, and MFF played pivotal roles in governing mitochondrial fission and remodeling MRC in HGG. The subsequent validation demonstrated that DNM1L/DRP1 exerts a positive regulatory effect on FIS1, whereas the interaction between MFF and FIS1 demonstrates a competitive inhibition relationship. The down-regulation of the DNM1L/DRP1-FIS1 axis significantly impaired mitophagy, thereby hindering the remodeling of MRC and inhibiting OXPHOS function in glioma, ultimately leading to the inhibition of its aggressive progression. In contrast, MFF exerts a contrasting effect on MRC integrity, OXPHOS activity, and glioma progression.

**Conclusions:**

This study highlights that the DNM1L/DRP1-FIS1 axis stabilizes MRC structures through mitophagy in HGG cells while driving their OXPHOS activity ultimately leading to robust disease progression. The inhibition of the DNM1L/DRP1-FIS1 axis hinders MRC remodeling and suppresses GBM progression. We propose that down-regulation of the DNM1L/DRP1-FIS1 axis could be a potential therapeutic strategy for treating HGG.

**Supplementary Information:**

The online version contains supplementary material available at 10.1186/s13046-024-03194-6.

## Introduction

The glioma is the most prevalent primary brain tumor, constituting 78.3% of malignant neoplasms in the central nervous system (CNS) [1].The fifth edition of the World Health Organization (WHO) Classification of Tumors of the Central Nervous System (WHO CNS 5) [2], published in 2021, categorized glioma into grade 1 – grade 4, with a progressive increase in malignancy. However, the dichotomization of low-grade glioma (LGG), encompassing grade 1 and grade 2, versus high-grade glioma (HGG), comprising grade 3 and grade 4, has garnered increased clinical interest. The median survival time for LGG typically exceeds 10 years [2], whereas the median survival time for HGG is merely around 2 years. The survival rate for patients with the highest HGG, glioblastoma (GBM), specifically, indicates that less than 75% of individuals survive beyond a duration of 13 months [3]. Therefore, it is urgent to explore the mechanism underlying high progression of HGG.

Previous studies implied that mitochondrial respiratory cristae (MRC) alterations exhibit a high degree of sensitivity towards the progression of malignant tumors [4]. Mitochondria have inner membrane invaginations called cristae that harbor functional respiratory complexes. MRC enhance efficiency of ATP production and accelerate nutrient oxidation by oxidative phosphorylation (OXPHOS). Dynamic of MRC and its OXPHOS outcome drive glioma progression [5–7], however, its mechanism remains unclear. Nearly a century ago, Otto Warburg first proposed that tumor cells derive energy not from OXPHOS provided by MRC, but rather from glycolysis, which exhibits a relatively lower rate of energy production [8]. The validity of this concept, however, has recently been called into question as it was found that OXPHOS is not inhibited as anticipated in the majority of cancer cells [9]. The findings of multiple studies have demonstrated that the mitochondria in various malignant tumor cells exhibit a propensity for excessive fission, as observed in lung cancer [10], liver cancer [11], pancreatic cancer [12], breast cancer [13] and glioma [14]. Previous studies have observed that cancer cells have hyper-activated MRC which contributed to invasive potential of malignant tumors [15, 16]. Recent studies have revealed that GBM can utilize astrocyte-derived mitochondria to enhance MRC, leading to the development of more aggressive tumors [17]. It should be noticed that the up-regulation of dynamin-1-like protein (DNM1L/DRP1) leads to an augmented MRC in BT25 and BT114 glioma stem cells, thereby facilitating the migration and invasion of glioma [18]. The different results imply the necessary to uncover the exact state of MRC and OXPHOS in HGG.

Currently, the focus of research on MRC reconstruction primarily revolves around two key aspects: elucidating alterations in its inherent structure and comprehending their consequential impact on its functionality. Firstly, an extensive body of literature has consistently demonstrated that heightened expression levels of proteins associated with mitochondrial division not only facilitate the process itself but also concurrently augment the abundance of MRC [19, 20]. Moreover, studies have been conducted to directly observe the proportion of the area occupied by MRC within the cross-section of mitochondria in order to assess their capacity for MRC remodeling [21, 22]. The MRC functions as a dynamic bioenergetic compartment and serves as the primary site for OXPHOS. Consequently, the enhancement of OXPHOS function is intricately linked to MRC remodeling [22–24]. The findings of various studies have demonstrated that mitochondria damage is characterized by the impairment of MRC, diminished respiratory activity and mitochondrial membrane potential (MMP), as well as alterations in gene expression and enzyme activity associated with reactive oxygen species (ROS) metabolism, ultimately resulting in an augmented accumulation of ROS within cells. Consequently, both MMP and ROS levels can serve as reliable indicators reflecting the status of MRC [25–28]. The protein PTEN induced putative kinase 1 (PINK1), located in the MMP, possesses the ability to detect mitochondrial damage and can be recruited to damaged mitochondria. This recruitment leads to the activation of PARKIN, an E3 ubiquitin ligase, resulting in the formation of a complex that repairs damaged mitochondria and reshapes the MRC. Consequently, there is a significant increase in MRC filling, ultimately enhancing mitochondrial function [19, 21, 29, 30].

The goal of the present study is to explore the mechanism underlying the dynamic of MRC and its OXPHOS outcome driving HGG progression. First, machine learning-based transmission electron microscopy analysis of 7141 mitochondria from 54 resected patients’ glioma, combined with bioinformatics analysis and multiplex immunohistochemical (mIHC) staining of clinical glioma microarrays, showed that, HGG exhibits a more prominent mitochondrial fission trend and a more integrated MRC compared to LGG. DNM1L/DRP1 and its two receptors mitochondrial fission protein 1 (FIS1) and mitochondrial fission factor (MFF) were screened as the key modulators for MRC in HGG. Followed virus-based regulation studies and the tests of mitochondrial function and glioma feature showed that targeted down-regulation of the DNM1L/DRP1-FIS1 axis in GBM cells can effectively inhibit mitophagy, impede the remodeling of MRC, result in a significant reduction in OXPHOS function, thereby exerting inhibitory effects on the progression of HGG. Additionally, another DNM1L/DRP1 receptor MFF exerted opposite role in MRC, OXPHOS and glioma progression. We propose that down-regulation of DNM1L/DRP1-FIS1 axis could be used for cure of HGG.

## Materials and methods

### Study strategy

As shown in Supplementary Fig. 1, we carried out the fine study focusing on MRC and its OXPHOS outcome in HGG from three aspects. First in green was to resolve morphological feature of MRC situation in HGG, by using machine learning-based transmission electron microscopy analysis of 7141 mitochondria from 54 resected patients’ glioma and to screen the key genes in it, by using bioinformatics analysis and mIHC staining of clinical glioma microarrays. Second in blue, lentivirus technology was employed to target and regulate DNM1L/DRP1 and its receptors FIS1 and MFF. The impact of these molecules on mitochondrial division and fusion proteins, as well as their morphological structures, was assessed using Western blot and electron microscopy techniques. Additionally, in conjunction with Seahorse XF technology, ROS detection, MMP detection, and MRC abundance measurement were utilized to investigate the effects of DNM1L/DRP1, FIS1, and MFF on the morphological structure (MRC abundance) and function (OXPHOS) of MRC in U87MG, U251MG, and GSCs. Furthermore, the influence of DNM1L/DRP1 and its receptors FIS1 and MFF on mitophagy was analyzed using Western blot technology along with Mito-Keima technology. Third in pink was to verify the therapeutic effects of blocking DNM1L/DRP1-FIS1 on HGG progression through remodeling MRC, by using CCK-8, Transwell, Wound Healing, tumor spheroid formation assay and cell derived xenograft assay.

### Acquisition of glioma clinical samples

Glioma tissue samples from 54 patients were collected from the Department of Neurosurgery of the First Affiliated Hospital of The Fourth Military Medical University from June 2021 to July 2023 (Clinical information was provided in Table 1). The collection of human glioma samples from the First Affiliated Hospital of The Fourth Military Medical University were approved by the ethics committee of the hospital (Human glioma sample ethical approval number: KY20232288-C-1). The clinical samples of all included patients were confirmed as glioma by pathological diagnosis and molecular examination, and the clinical and pathological data were complete. The collected tissue specimens were promptly removed from the body within 5 min, ensuring they remained on ice at a temperature of 4℃. Subsequently, the core tissues were precisely divided into three equal pieces measuring 1 mm × 1 mm × 1 mm each. These sections were then immersed in Gluta fixation solution (P1126, Solarbio, China) for electron microscopy and stored at -80℃.

**Table 1:** Baseline characteristics of the patients

Characteristics	Total (*n* = 54)
Sex-no. (%)	
Male	35 (64.8)
Female	19 (35.2)
Age-yr	
Median	47
Range	23—81
WHO Grade-no. (%)	
Grade 1	4 (7.4)
Grade 2	11 (20.4)
Grade 3	15 (27.8)
Grade 4	24 (44.4)
IDH Mutations-no. (%)	
IDH1	25 (46.3)
IDH2	1 (1.9)
Wild Type IDH	26 (48.1)
Missing data	2 (3.7)
Tumor Classification-no. (%)	
Pilocytic Astrocytoma	4 (7.4)
Astrocytoma, IDH-mutant	10 (18.5)
Oligodendroglioma, IDH-mutant and 1p/19q co-deletion	16 (29.6)
Glioblastoma, IDH-wide type	24 (44.5)

### Transmission electron microscope of clinical samples

The human brain glioma samples stored at -80℃ were retrieved and transferred onto ice at 4℃. Following the previously reported transmission electron microscope preparation method [31], the tissues were initially rinsed with 0.1 mol/L phosphate buffer saline (PBS) for three cycles of 5 min each. Subsequently, the tissues were fixed in a light-protected environment at 4℃ using 1% urea acid for two hours, followed by another round of washing with 0.1 mol/L PBS for three cycles of 5 min each. The samples were then dehydrated in 50% ethanol, 70% ethanol and 90% ethanol for 15 min each. Then the samples were dehydrated in a 1:1 mixture of acetone and embedding agent for 60 min. After overnight in pure embedding agent, the samples were embedded in a new embedding agent and fixed in a resin plate, and then dried for 48 h in an oven at 68°C. The 50 nm sections were collected on a copper mesh and stained with lead nitrate and uranyl acetate for 10 min. Images were taken by transmission electron microscopy (Jeol 1230, JEOL, Japan). Only the electron microscopy images that encompassed the entire cell membrane field in a single photo were chosen for the morphological analysis of mitochondria in tumor cells.

### Machine learning of transmission electron microscopic images

Mitochondrial morphology was analyzed by ImageJ (1.53c, NIH, USA). Transmission electron microscope pictures of the intact cell membrane field were selected and opened in this software. We Selected “Set Scale” in “Analyze” of the taskbar, and converted the measurement unit to “μm” according to the scale in the transmission electron microscope picture. Then mark the cell membrane, nucleus and mitochondria. The cell membrane, nuclear membrane and outer mitochondrial membrane (OMM) were outlined by clicking one by one, and then clicked the “ctrl” + “m” to read the morphological parameters of cells and mitochondria.

We utilized Mask Region-based Convolutional Neural Network (Mask R-CNN), a typical instance segmentation model, to accurately detect mitochondria in the given image. Mask R-CNN, proposed by Kaiming He et al. [32], is widely recognized as a prominent model employed for object detection and instance segmentation tasks. This model comprises three output branches: one dedicated to determining the precise bounding box coordinates, another responsible for predicting the specific class of each instance, and the third focused on generating accurate masks corresponding to each individual instance. Through end-to-end training, Mask R-CNN could acquire the discriminative features of mitochondria, enabling precise pixel-level identification of individual mitochondria in input images. To enhance the model's ability to recognize mitochondria, we curated a novel dataset comprising 50 training images and 10 test images captured by electron microscopy. Each image has a resolution of 2048 × 2048 pixels, with manual pixel-level annotations for instances about mitochondria. In comparison to the widely-used Common Objects in Context (COCO) dataset for instance segmentation [33], our constructed database was relatively small in terms of image quantity and more susceptible to overfitting. To address this situation, we employed pre-training on the COCO dataset to enhance our model's performance and evaluated the impact of COCO pre-training on our experimental outcomes. For this purpose, we conducted a comparative experiment: one group utilized Mask R-CNN weights that were pre-trained on the COCO dataset and then fine-tuned on our constructed dataset, while the other group directly trained on our built dataset without any pre-training. The training and fine-tuning setup remained identical for both groups. ResNeXt-101 (64 × 4d) was adopted as the backbone architecture of Mask R-CNN. During the training (fine-tuning) process, we began by resizing each input image using a randomly selected scale from [640, 672, 704, 736, 768, 800]. Subsequently, to mitigate overfitting effects, we applied random image flipping before feeding it into the model. For testing purposes, the test images were resized to dimensions of 800 × 800 pixels and then fed into the model. We trained the model using stochastic gradient descent (SGD) for 30 k iterations, with a mini batch size of 1. The initial learning rate was set to 0.02, momentum to 0.9, and weight decay to 0.0001. For training and fine-tuning, we utilized a dataset consisting of 50 transmission electron microscopy images. Following the training (fine-tuning) process, we evaluated the model's performance on instance segmentation by inputting 10 test images sequentially and comparing the prediction results with manual labels. The evaluation criteria used were COCO evaluation metrics, including Bbox AP and Segm AP. The Pytorch architecture was utilized for the implementation of the model, and both training and testing were conducted on a PC equipped with Tesla V100 32 GB. Once the recognition algorithm met the current requirements, individual intracellular mitochondria in transmission electron microscope images with a resolution of 5 k were identified. A total of 54 human glioma samples and 276 complete cell transmission electron microscope images were employed to identify a total of 7141 mitochondria within the cells.

In the process of measuring mitochondrial morphological indicators, only cells with intact cell membranes were selected as a standardized criterion for measuring all intracellular mitochondrial morphological indicators. For mitochondria with uncertain determinability, high magnification was employed for assessment and determination. Among N test images, instances with a model prediction score < 0.5 in each image were discarded, while instances with a score ≥ 0.5 and any ground truth IoU ≥ 0.5 in the prediction were considered true positives (TP) (indicating high confidence and accurate localization). Instances with a score ≥ 0.5 but each ground truth IoU < 0.5 were counted as false positives (FP) (indicating high confidence but inaccurate localization), and the remaining ground truths that did not match correctly were counted as false negatives (FN). The definitions of Precision, Recall, and F1 score were as follows:

$$Precision=\frac{1}{N}(\frac{TP}{TP+FP})$$

$$Recall=\frac{1}{N}(\frac{TP}{TP+FN})$$

$$F1 score=\frac{1}{N}(\frac{TP}{TP+\frac{FN+FP}{2}})$$

The perimeter error rate and area error rate between the model-predicted mitochondria instances and ground truth were designed and compared for the first time, in addition to considering morphological indicators:

$$Perimeter\,error\,rate=\frac{1}{N}\sum\limits_{pred\_i {\epsilon}TP}\frac{P_{pred_i}-P_{gt\_i}}{P_{gt\_i}}$$

$$Area\,error\,rate=\frac{1}{N}\sum\limits_{pred\_i {\epsilon}TP}\frac{A_{pred_i}-A_{pred\_i}}{A_{pred\_i}}$$

${P}_{pred\_i}$ (${A}_{pred\_i}$) represented the perimeter (area) of the instances belonging to TP predicted by the model in each image, while ${P}_{gt\_i}$ (${A}_{gt\_i}$) denoted the perimeter (area) of the ground truth matching ${P}_{pred\_i}$ (${A}_{pred\_i}$).

The calculation indices encompassed the following five parameters: 1) mitochondrial density, represented by the number of mitochondria per unit cell area; 2) individual mitochondrial size, indicated by the area of a single mitochondrion; 3) individual mitochondrial shape, quantified by the perimeter of a single mitochondrion; 4) relative mitochondrial size, expressed as the proportion of mitochondrial area to unit cell area; 5) mitochondrial morphology, characterized by the circularity of a single mitochondrion. Among these indices, parameters 1)—3) pertained to individual mitochondrial size while parameters 4) and 5) described its morphology.

According to the classification method proposed by Mingqi Han et al. [4], our study categorized the mitochondria in human brain glioma samples collected clinically into three types based on the organization of MRC and the integrity of OMM: Type 1 exhibited dense and orderly arranged MRC with intact OMM; Type 2 displayed sparse and disorganized MRC or a disrupted OMM; Type 3 showed either vanished MRC or combined with ruptured OMM.

### Cultured GBM cell lines

GBM cell lines U87MG (CL-0238) and U251MG (CL-0237), which were purchased from Procell Life Science & Technology Co., LTD., were identified by Short Tandem Repeat (STR) analysis. Minimum Essential Medium (MEM) (C11995500BT, Gibco, USA) was used for U87MG, and Dulbecco's Modified Eagle's Medium (DMEM) (C11995500BT, Gibco, USA) was used for U251MG, and both were cultured with 10% fetal bovine serum (FBS) (164210-50, Procell, China) and 1% penicillin/streptomycin (PB180120, Procell, China). Cells were placed in an incubator at 37°C, 5% CO_2_ and 100% relative humidity and were routinely sub-cultured. All cells tested negative for mycoplasma.

The GSCs were generously provided by Professor Guo Qingdong's team from the Neurosurgery Department of Xijing Hospital, The Fourth Military Medical University. These cells were derived from human GBM samples that met the hospital's ethical guidelines and transfer agreement. The collection and transfer of biological samples strictly adhered to the hospital's relevant agreements. Prior to experimentation, GSCs were assessed for stem cell-specific markers CD133 and SRY-related HMG-box 2 (SOX2) using immunofluorescence staining to confirm their stem cell identity and meet predetermined criteria. To optimize the in vitro culture environment of GSCs, they were cultured and passaged in CTS Neurobasal medium (Gibco, A13712-01) supplemented with 1% penicillin/streptomycin solution to prevent microbial contamination, as well as 10% FBS, based on a review of relevant literature [34–36]. In addition, growth factors and nutritional additives were supplemented: 2 mmol/L L-glutamine (Gibco, 25030081) to enhance cell growth and metabolism, 2 mmol/L N-2 additive (100X) (Gibco, 17502048) to provide essential nutrients, 1% non-essential amino acid (NEAA) (Gibco, 11140050) to support cellular anabolism, 2% B-27™ Additive (50X) serum-free (Gibco, 17504044), a supplement designed for neurons and neural stem cells; along with the synergistic promotion of cell proliferation and self-renewal by adding 20 ng/mL basic fibroblast growth factor (bFGF) (MCE, HY-P7004) and 20 ng/mL epidermal growth factor (EGF), (MCE, HY-P7109). The incubator was set at a condition of 37°C with a CO₂ level of 5% to ensure normal cell growth and metabolism. Regular monitoring of changes in cell morphology, proliferation status and culture medium color was conducted to promptly adjust the culture conditions.

### Overexpression and interference lentiviruses of DNM1L/DRP1, FIS1 and MFF

The lentiviral transfection was packaged and synthesized by Hanbio Biotechnology Co., LTD. After the vector plasmid carrying the target gene or shRNA, the virus packaging helper plasmid (psPAX2 vector), and the virus packaging helper plasmid (pMD2G vector) were extracted with high purity and without endotoxin, the three plasmids were co-transfected into 293 T cells using LipofiterTM transfection reagent from Hanbio (lentiviruses information was provided in Table 2). The virus supernatant was collected at 48 h and 72 h after transfection, packaged in the virus kit, and stored at -80°C. U87MG/U251MG/GSCs in good condition were inoculated on 6-well plates for lentivirus transfection [37]. At 48 h after infection, the efficiency of green fluorescent protein (GFP) expression was initially observed by fluorescence microscopy. The optimal multiplicity of infection (MOI) of lentivirus transfection into U87MG/U251MG/GSCs was 5. The screening concentration of puromycin was 2 µg/mL [38]. Experiments should be conducted after stable transfection for two weeks.

### Mitochondrial division inhibitor Mdivi-1

**Table 2:** Lentivirus used in the study

Lentivirus	Groups	Vendor	Item No	Batch No	Lentivirus vector	Titer (TU/mL)
DNM1L/DRP1	sh-DNM1L-NC	Hanbio	HH20221001ZY-LV01	LV72101843	pHBLV-U6-MCS-CMV-ZsGreen-PGK-PURO	3*10^8
DNM1L/DRP1	sh-DNM1L	Hanbio	HH20221001ZY-LV01	LV72101844	pHBLV-U6-MCS-CMV-ZsGreen-PGK-PURO	3*10^8
DNM1L/DRP1	OE-DNM1L-NC	Hanbio	HH20221001ZY-LP01	LV72101416	pHBLV-CMV-MCS-3FLAG-EF1-ZsGreen-T2A-PURO	3*10^8
DNM1L/DRP1	OE-DNM1L	Hanbio	HH20221001ZY-LP01	LV72101417	pHBLV-CMV-MCS-3FLAG-EF1-ZsGreen-T2A-PURO	2.5*10^8
FIS1	sh-FIS1-NC	Hanbio	HH20230505ZY-LV01	LV79051633	pHBLV-U6-MCS-CMV-ZsGreen-PGK-PURO	1.5*10^8
FIS1	sh-FIS1	Hanbio	HH20230505ZY-LV01	LV79051635	pHBLV-U6-MCS-CMV-ZsGreen-PGK-PURO	2*10^8
FIS1	OE-FIS1-NC	Hanbio	HH20230505ZY-LP01	LV79050901	pHBLV-CMV-MCS-3FLAG-EF1-ZsGreen-T2A-PURO	1.5*10^8
FIS1	OE-FIS1	Hanbio	HH20230505ZY-LP01	LV79050902	pHBLV-CMV-MCS-3FLAG-EF1-ZsGreen-T2A-PURO	4*10^8
MFF	sh-MFF-NC	Hanbio	HH20230822ZY-LV01	LV83090134	pHBLV-U6-MCS-CMV-ZsGreen-PGK-PURO	1.5*10^8
MFF	sh-MFF	Hanbio	HH20230822ZY-LV01	LV83090132	pHBLV-U6-MCS-CMV-ZsGreen-PGK-PURO	1.5*10^8
MFF	OE-MFF-NC	Hanbio	HH20230822ZY-LP01	LV82082903	pHBLV-CMV-MCS-3FLAG-EF1-ZsGreen-T2A-PURO	2*10^8
MFF	OE-MFF	Hanbio	HH20230822ZY-LP01	LV82082904	pHBLV-CMV-MCS-3FLAG-EF1-ZsGreen-T2A-PURO	2*10^8
Mito-Keima	-	Hanbio	HH20240621ZY-LV01	AP24041002	-	3.16*10^10

5 mg of Mdivi-1 (M0199, Sigma, USA) powder was dissolved in 0.283 mL Dimethyl sulfoxide (DMSO) (D2650, Sigma, USA) and configured into a 50 mmol/L Mdivi-1 solution. 50 µL was added to 1.95 mL of serum-free DMEM (C11995500BT, Gibco, USA) medium to make 2 mL of 1.25 mmol/L solution, filtered to remove bacteria, and 100 µL of filtered Mdivi-1 solution was added to each 5 mL of medium. The concentration was 25 µmol/L. At the same time, 2.5 µL of DMSO was added per 5 mL of medium in the control group. The Mdivi-1 drug group and DMSO group were treated for 2 h.

### Oxygen consumption rate (OCR) and extracellular acidification rate (ECAR) determination

According to the manufacturer's protocol, we measured OCR and ECAR on the Seahorse Real-time Cell Metabolism Analyzer (Seahorse XFe96, Agilent, USA) using the Seahorse XF Cell Mitochondria Stress Test Kit (103015-100, Agilent, USA) and the Seahorse XF Glycolysis Rate Assay Kit (103344-100, Agilent, USA), respectively. U87MG and U251MG successfully transfected with DNM1L/DRP1 lentivirus, FIS1 lentivirus and MFF lentivirus were seeded into 96-well plates (103794-100, Agilent, USA) at a density of 5 × 10^4^ cells/well and incubated overnight. After washing the cells with Seahorse buffer, oligomycin (1.5 µM), followed by Carbonyl cyanide 4-(trifluoromethoxy) phenylhydrazone (FCCP) (1.5 µM), and finally rotenone (0.5 µM) and antimycin (0.5 µM) were automatically injected to measure OCR. Another 96-well plate was seeded with the same number of cells and then glucose, oligomycin, and 2-deoxyglucose (2-DG) were added to determine ECAR. OCR and ECAR values were calculated for each group of proteins after normalization using Seahorse Wave Desktop (2.6.3.5, Agilent, USA) with five samples per group for statistical analysis. The OCR/ECAR was calculated from the normalized OCR value and EACR value of each well according to the corresponding method of the same treatment and the same group, and then grouped for statistical analysis. U87MG and U251MG treated with 25 mmol/L Mdivi-1 or 0.1% DMSO for 2 h were used in the same way.

### MMP detection

Preparation of MMP Assay Kit (TMRE) (C2001S, Beyotime, China) dyeing solution. U87MG and U251MG successfully transfected with DNM1L/DRP1 lentivirus, FIS1 lentivirus and MFF lentivirus were digested into cell suspension with 0.25% trypsin. Centrifuged appropriate number of cells at 600 × g for 5 min at room temperature, and removed the supernatant. Then resuspended the cells by adding 500 µL of TMRE staining solution, aiming for a cell density of approximately 1 × 10^6^/mL. Set the positive control at the same time: the Carbonyl cyanide 3-chlorophenylhydrazone (CCCP) (10 mmol/L) provided in the kit was recommended to be added to the cell culture medium at a ratio of 1:1000 and diluted to 10 µmol/L. Six samples from each experimental group were analyzed by flow cytometry (FACS Celesta, BD, USA). The test readiness should be ensured by maximizing the sufficiency of each group of cells and conducting the test expeditiously to prevent sample fluorescence quenching. The maximum excitation wavelength of TMRE was 550 nm and the maximum emission wavelength was 575 nm. FlowJo10.8.1 was used for analysis.

### ROS detection

According to the instructions of the mitochondria ROS Assay Kit (C10491, Thermo Fisher, USA): U87MG and U251MG successfully transfected with DNM1L/DRP1 lentivirus, FIS1 lentivirus and MFF lentivirus were digested with 0.25% trypsin to make cell suspension. An appropriate number of cells were centrifuged at 600 × g for 5 min at room temperature. Cell staining was performed by 1 µL of cell permeable CellROX®Deep Red reagent. Tert-Butyl Hydroperoxide (TBHP) was used as a positive control, and N-acetyl-L-cysteine (NAC) was used as a negative control. Six samples from each experimental group were sequentially analyzed by flow cytometry after 40 min of incubation. The maximum excitation wavelength was 644 nm and the maximum emission wavelength was 665 nm during flow cytometry (B75442, Beckman Coulter, USA). FlowJo10.8.1 was used for analysis.

### Transmission electron microscope preparation of cultured cells

U87MG were divided into groups according to Mdivi-1, DMSO, DNM1L/DRP1 lentivirus, FIS1 lentivirus, and MFF lentivirus transfection, and each group had 3 dishes of cells. The culture medium of the dishes was removed and 2-3 mL of 2.5% Gluta fixative (P1126, Solarbio, China) that had been restored to room temperature was added. After the cells were fixed at room temperature in the dark for 5 min, to avoid scraping and destroying cells, they were quickly shoveled off in one direction at an oblique 45° with a cell scraper. The resulting cell suspension was aspirated into a centrifuge tube and centrifuged at 3000 rpm for 5 min. The supernatant was discarded and a new 2.5% Gluta fixative specific for electron microscopy was added. After fixation at room temperature in the dark for 30 min, the plates were stored at 4°C. The methods of sample preparation, filming, measurement and statistics were consistent with the methods of glioma sample processing. In order to avoid the swelling and deformation of cells and sub-organelles, the electron microscopy should be made within 7 days.

### Western blot

To confirm the successful intervention of DNM1L/DRP1, FIS1, MFF lentivirus and Mdivi-1 in U87MG, U251MG and GSCs, total proteins were extracted, the protein concentration was normalized and Western blot analysis was performed to detect the expression of DNM1L/DRP1, FIS1 and MFF. Meanwhile, the expression levels of p-DNM1L^Ser616^, p-DNM1L^Ser637^, MFN2, SOD1, PINK1, PARKIN, ATG5, LC3B, p62, NIX and FUNDC1 in GBM cells treated with DNM1L/DRP1, FIS1 and MFF lentivirus were detected (Table 3 for antibody information). They were detected with enhanced chemiluminescence (SF009, SAIFEISI, China) followed by exposure to a photometer (Fusion FX6-XT, VIBER, France) and analyzed with ImageJ (1.53c, NIH, USA). Target protein levels were normalized relative to the level of the reference protein β-Actin and expressed as fold change relative to the initial control.

### Immunofluorescent staining

**Table 3:** Antibodies used for WB (Western blot) / IF (immunofluorescence) / mIHC (multiplex immunohistochemistry)

Antibody	Source	Vendor	Dilution	Cat. #	WB/IF/mIHC
ATG5	Rabbit	ABclonal	1:1000	AP19677	WB
β-Actin	Rabbit	Servicebio	1:1000	DB 1013	WB
CD133	Rabbit	Servicebio	1:800	GB114579-100	IF
DNM1L/DRP1	Rabbit	Cell signaling Technology	1:1000	8570	WB
DNM1L/DRP1	Rabbit	Abcam	1:1000	ab184247	mIHC
p-DNM1L ^Ser616^	Rabbit	ABclonal	1:1000	AP0849	WB, mIHC
p-DNM1L ^Ser637^	Rabbit	ABclonal	1:1000	AP0812	WB, mIHC
FIS1	Rabbit	Abcam	1:1000	ab156865	WB, mIHC
FUNDC1	Rabbit	ABclonal	1:1000	A16318	WB
HRP	Mouse	Abbkine	1:5000	A21010	WB
HRP	Rabbit	Abbkine	1:5000	A21020	WB
Ki-67	Rabbit	Servicebio	1:500	GB151142-100	IF
LC3B	Rabbit	Abcam	1:1000	ab192890	WB
MFF	Mouse	Santa	1:1000	sc-541077	mIHC
MFF	Rabbit	Cell signaling Technology	1:1000	84,580	WB
MFN2	Rabbit	Cell signaling Technology	1:1000	9482	WB
NIX	Rabbit	Abcam	1:1000	ab155010	WB
p62	Rabbit	Cell signaling Technology	1:1000	8025S	WB
PARKIN	Rabbit	Invitrogen	1:1000	PA5-13,399	WB
PINK1	Rabbit	Invitrogen	1:1000	PA5-85,930	WB
SOD1	Rabbit	ABclonal	1:1000	A0274	WB
SOX2	Rabbit	Servicebio	1:500	GB11249-100	IF

U87MG, U251MG, and GSCs were individually seeded onto pre-treated confocal dishes to achieve an optimal cell density for subsequent observation. Upon reaching approximately 60% confluence, the cells were transfected with Mito-Keima adenovirus. Following a 48-h incubation period, the cells were gently washed thrice with pre-cooled PBS to eliminate residual culture medium and unbound virus particles, with each wash lasting 5 min. Subsequently, a 4% paraformaldehyde (PFA) solution was applied to fix the cells at room temperature for 30 min in order to preserve their morphology and cellular structures. After fixation, the cells underwent another three rounds of PBS washing for complete removal of residual PFA. The fixed and washed cell samples were then subjected to high-resolution imaging of mitochondria using a Leica confocal microscope (Leica STELLARIS 5) at 440 nm and 561 nm channels. Quantitative analysis of the fluorescence images was conducted using ImageJ.

The GSCs were inoculated into pre-treated confocal dishes at an optimal cell density for subsequent observation. Upon reaching approximately 80% confluency, the cells were gently washed three times with pre-cooled PBS for 5 min each to eliminate residual culture medium. Subsequently, the cells were fixed in a 4% PFA solution at room temperature for 30 min to preserve their morphology and cellular structure. Following fixation, the cells underwent three washes with PBS to thoroughly remove any remaining PFA. Next, a blocking solution of PBS containing 1% BSA was added to the confocal dish and incubated at room temperature for 30 min. After discarding the blocking solution, CD133 antibody (diluted at a concentration of 1:1000) and SOX2 antibody (diluted at a concentration of 1:500) were applied evenly across the entire surface of the dish according to the experimental protocol provided by the reagent supplier. The confocal dish was then placed in a humidified chamber and incubated overnight at 4°C before removing the antibody solution and washing the cells three times with PBS for 5 min each to eliminate unbound antibodies. The corresponding secondary antibody was subsequently incubated in darkness at room temperature for one hour, followed by another round of washing with PBS three times. DAPI was then introduced into the confocal dish to stain and label cell nuclei, followed by incubation at room temperature for 5-10 min. After removing excess DAPI solution, cells were washed twice with PBS for five minutes each. Finally, specimens were fixed and mounted using anti-fluorescence mounting medium before imaging on a Leica STELLARIS5 confocal microscope; analysis was performed using ImageJ.

In accordance with the experimental protocol and animal welfare guidelines, tumor-bearing mice were promptly subjected to aseptic surgical procedures for the extraction of tumor tissue upon reaching the predetermined stage of tumor growth. The excised tumor samples were immediately immersed in pre-chilled physiological saline on ice for a brief rinse to eliminate excess blood and impurities. Subsequently, the samples were fixed in 4% PFA at 4°C overnight to preserve tissue architecture and protein conformation. Following completion of fixation, the samples underwent thorough washing with PBS three times, each time for 10 min, to remove residual PFA. Thereafter, they were transferred to a 20% sucrose solution and further dehydrated at 4°C for 48 h to enhance tissue rigidity and transparency in preparation for subsequent sectioning. The dehydrated tumor tissue was then sliced into 30-micron-thick sections using a cryostat. These sections were air-dried and incubated at room temperature with FBS for 30 min to minimize non-specific binding of antibodies within the tissue and reduce background fluorescence. Upon removal from the blocking solution, a primary antibody (Ki-67, R, 1:500) was directly applied and incubated in a humidified chamber at 4°C overnight (approximately16 hours). Subsequent washing with PBS three times at room temperature for 5 min each effectively removed unbound primary antibody. The secondary antibody was then incubated at room temperature in darkness for 1 h, followed by triple washes with PBS before staining with DAPI to label cell nuclei. After mounting and drying the specimens using anti-fluorescence mounting medium, the images were captured using a Leica confocal microscope (Leica STELLARIS5) and analyzed utilizing ImageJ.

### CCK-8 assay

U87MG, U251MG and GSCs successfully intervened by DNM1L/DRP1 lentivirus, FIS1 lentivirus and MFF lentivirus were inoculated at a density of 5 × 10^3^ cells/well in 200 μL DMEM medium in 96-well plates, with 6 cell samples in each group [35]. Note that air bubbles should be avoided as much as possible in the process of sample addition, and the culture plate was placed in the incubator for 2 h. After 2 h (after cell attachment), 24 h, 48 h and 72 h of culture, 10 μL CCK-8 reagent (CA1210-500 T, Solarbio, China) was added to each well, and then the cells were cultured in the original medium for another 2 h. After that, the absorbance at 450 nm was calculated using a microplate reader to obtain cell viability.

### Wound Healing assay

U87MG and U251MG successfully interfered with DNM1L/DRP1 lentivirus, FIS1 lentivirus and MFF lentivirus were digested and resuspended, then inoculated in 6-well plates (5 × 10^5^ cells/well). After observing that the confluence of cells reached about 90%, the cells were scraped with a 1 ml blue pipette tip in a horizontal line at a uniform speed. The crossed-out cells were washed and added in serum-free blank DMEM mediums. The cells were placed in a 5% CO_2_ incubator at 37°C and observed under a microscope (CKX53, OLYMPUS, Japan) at 0 h, 12 h, and 24 h and photographed. ImageJ (1.53c, NIH, USA) was used to measure the area of cell migration, and to analyze the ability of cell migration. The calculation was as follows: the ratio of 24 h migration area (scratch area at 0 h-scratch area at 24 h) to 0 h area.

### Transwell assay

The 6.5 mm diameter inserts, 8.0 μm pore size cross-pore chambers (3422, Costar, USA) were placed in 24-well plates with 200 μL of low-serum DMEM medium added to the upper layer and 500 μL of DMEM medium added to the bottom chamber [39]. U87MG and U251MG successfully interfered with DNM1L/DRP1 lentivirus, FIS1 lentivirus and MFF lentivirus were digested, resuspended, inoculated in the upper chamber at a density of 5 × 10^5^/ chamber, then cultured at 37℃, 5% CO_2_ and 100% relative humidity for 48 h. The medium was removed and the cells were washed twice with PBS before being fixed with 4% PFA (R20497, Shyuanye, China) and stained with 0.1% crystal violet solution (C0121, Beyotime, China) for 15 min. After washing with PBS for three times, the inner side of the chamber was carefully wiped with a cotton swab to remove the residual cells to avoid interfering the experimental results. Cell migration was observed under a microscope (CKX53, OLYMPUS, Japan) at 10 × . Finally, ImageJ (1.53c, NIH, USA) was used to count the number of migrated cells.

### Tumor spheroid formation assay

After virus transfection, stable GSCs were seeded at a density of 3 × 10^3^ cells/mL in Corning® Costar® ultra-low attachment 6-well plates (Corning, CLS3471) and gently agitated to ensure even distribution. The CTS Neurobasal culture medium was prepared according to the optimized formula, with the addition of growth factors and nutrient supplements as mentioned earlier. The culture plate was then placed in an incubator at 37°C with 5% CO₂ saturated humidity to facilitate neural ball formation. Every three days, the morphology of the neural balls was observed under an inverted microscope and recorded. On day 10 post-inoculation, images of neural balls in each well were captured using an inverted microscope. These images were analyzed using ImageJ for diameter measurement through suitable threshold and edge detection algorithms. Subsequently, the volume of each neural ball was calculated based on its diameter. Statistical recording of the number of neural balls per well enabled calculation of average or total volume to evaluate efficiency in formation and growth. Data on number, diameter, and volume for each well were documented.

### Bioinformatics analysis of MRC-related genes

The RNA-seq data of GBM (*n* = 168) and LGG (*n* = 529) were download from The Cancer Genome Atlas (TCGA) [40] database. The RNA-seq data of normal brain tissues (*n* = 1152), including amygdala, anterior cingulate cortex (BA24), caudate (basal ganglia), cerebellar hemisphere, cerebellum, cortex, frontal cortex (BA9), hippocampus, nucleus accumbens (basal ganglia), putamen (basal ganglia), spinal cord (cervical c-1) and substantia nigra were collected from Genotype-Tissue Expression (GTEx) [41] database. The mitochondria-related genes (MRGs) were obtained from MitoCarta3.0 [42]. The differential expression analysis was conducted between the tumor (GBM/LGG) and normal brain tissues by “Limma” package [43]. The screening criteria utilized for MRGs are: adjusted *P* < 0.05 and |log2 (Fold Change) |> 0.585. The R package "ggplot2" based on R programming language (versions R-4.1.3) was used to data visualization (Specific information was provided in Supplementary Table 1).

### mIHC staining of microarrays

Human glioma tissue microarrays HBraG125PG01 and HBra-Gli060PG-01 were purchased from Shanghai OUTDO BIOTECH Co., LTD. There were 122 glioma spots and 3 normal brain tissue spots on HBraG125PG01. HBra-Gli060PG-01 included 57 glioma spots and 3 normal brain tissue spots. The two microarrays had 185 spots in total, but some were missing. After H&E staining, the defective parts were eliminated, and 170 spots were left (Microarray information was provided in Supplementary Table 2).

According to the protocol provided by the manufacturer [44–46], the staining process consisted of 11 steps: baking sheet, dewaxing, antigen repair, removal of endogenous peroxidase, blocking and removal of slides, primary antibody incubation, secondary antibody incubation, color development with Opal dye (NEL801001KT, PerkinElmer, USA), microwave treatment to remove primary antibody and secondary antibody, counterstained indicators, DAPI staining, and dropping anti-fluorescence quenching sealant (H-1400, Vector Labs, USA) for seal sheeting. The stained microarrays were analyzed one by one using an automatic quantitative pathological imaging system (Vectra Polaris, PerkinElmer, USA). DAPI staining was used as the reference value for the number of cells in the same tissue, and the number of cells expressing five different proteins (p-DNM1L^Ser616^, p-DNM1L^Ser637^, DNM1L/DRP1, FIS1, MFF) was used as the number of positive cells. The percentage of positive cells was calculated and statistically analyzed.

### Cell derived xenograft assay in mice

We selected healthy male NOD/ShiLtJGpt-*Prkdc*^em26Cd52^*Il2rg*^em26Cd22^/Gpt (NCG) mice, aged between 4 - 6 weeks, utilizing six mice per group and establishing multiple experimental groups (including DNM1L-OE, DNM1L-KD, FIS1-OE, FIS1-KD, MFF-OE, MFF-KD, OE-NC, KD-NC) to comprehensively investigate the effects of various mitochondrial dynamics-related genes on glioma growth. Initially, we employed lentiviral vectors for stable transfection of target genes into two cell lines: the human glioma cell line U87MG-serving as a representative traditional glioma model-and GSCs, which provided a more cellular model closely resembling tumor origin and heterogeneity. We identified cell lines stably expressing the target genes through puromycin selection. Subsequently, we suspended the selected U87MG and GSCs at a concentration of 5 × 10^8^ cells/mL and injected 100 μL of this suspension (approximately 5 × 10^7^ cells) subcutaneously on the right dorsal side of each mouse. The condition of the nude mice was continuously monitored, and the subcutaneous tumor volume was measured on the 8th day when it reached a size between 50 - 100 mm^3^. Tumor length (L) and width (W) were measured every three days to calculate tumor volume (V = 0.5 × L × W × W). The experimental protocol was approved by the Laboratory Animal Welfare and Ethics Committee of The Fourth Military Medical University (animal experiment ethics approval number: IACUC-20231262). Throughout the course of the experiment, we adhered rigorously to the guidelines established by the Laboratory Animal Welfare and Ethics Committee of the The Fourth Military Medical University to ensure optimal welfare for the mice while monitoring tumor growth. We maintained that tumor weight did not exceed 10% of each mouse's body weight, with an average tumor diameter limited to no more than 16 mm, and ensured that tumor volume remained below 1500 mm^3^. Should any experimental mouse experience a weight loss exceeding 20% or develop ulcers resulting in infection or necrosis, the experiment would be terminated immediately, necessitating euthanasia of the animal. Ultimately, on completion day of the experiment, mice were euthanized via inhalation of 40% carbon dioxide, after which tumor tissues were promptly excised for subsequent analyses including molecular biology and histology.

### Statistical analysis

All statistics were measured using GraphPad Prism 8. For comparison, the t-test (for parametric data) and the Mann-Whitney U test (for nonparametric data) were used to determine statistical significance. Where multiple comparisons were made, one-way ANOVA test (for parametric data) or Kruskal-Wallis test (for nonparametric data) were used. Results were reported as the mean ± standard deviation (SD). All mIHC, Western blot, and cell function assays were performed ≥ 3 times to ensure reproducibility. *p* < 0.05 was considered statistically significant.

## Results

### Machine learning-based transmission electron microscopy analysis revealed compared with LGG, the mitochondrial fission trend in HGG is more significant and the MRC state is better

**Fig. 1:**
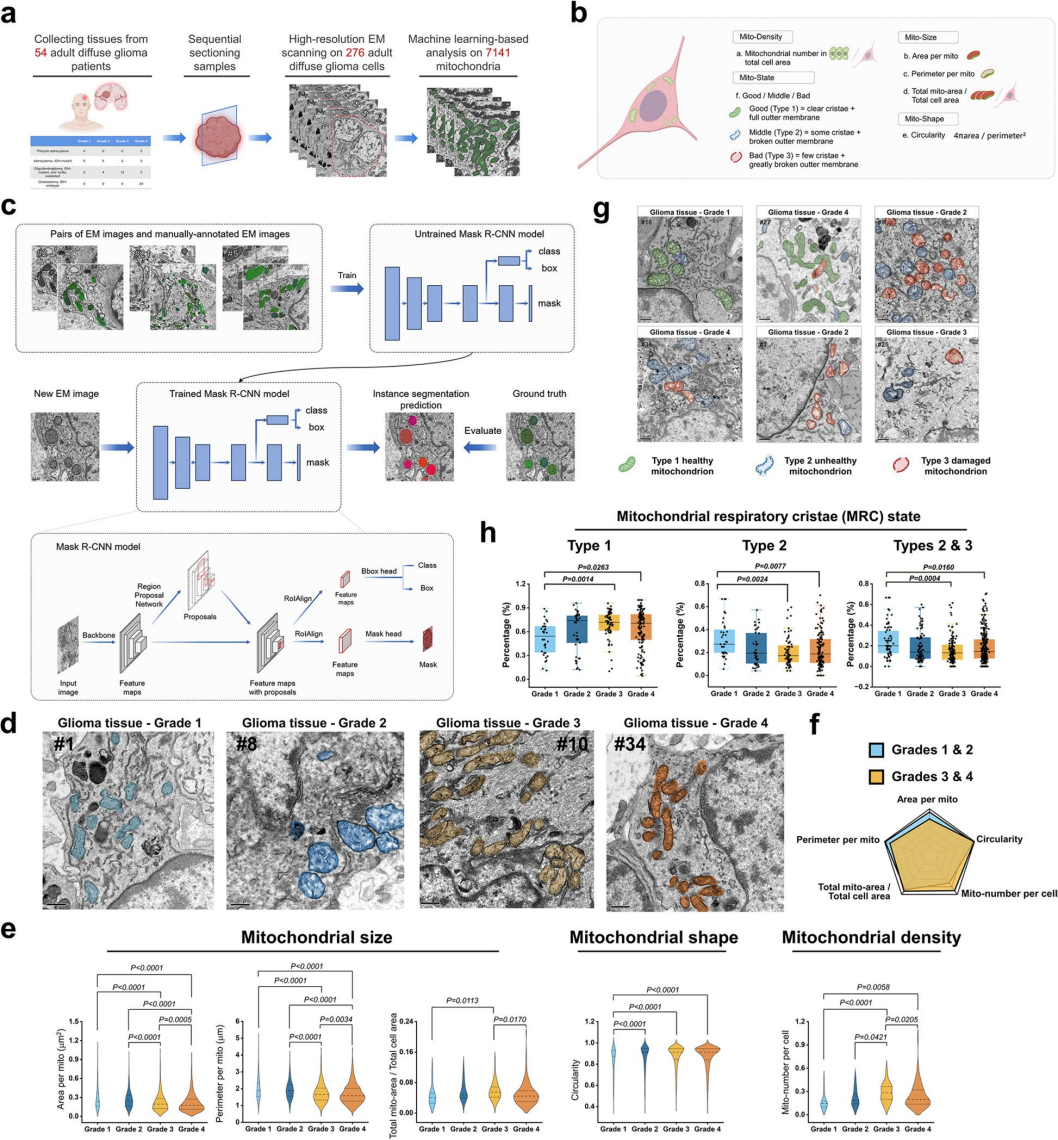
The identification and analysis of mitochondrial state in glioma under electron microscope by Machine Learning. **a **Analysis flow chart of a total of 7141 mitochondria of 54 glioma patients. **b** Mitochondrial state was valued in four aspects: **a**, Mito-density; **b** - **d**, Mito-size; **e**, Mito-shape; **f**, MRC state. **c** Mask R-CNN model for mitochondrial identification. **d** Electron microscopic representation of mitochondria in different grade glioma. Scale bars: 0.5 µm. **e** Statistical analysis of mitochondrial size, shape and density. Mitochondrial number were: Grade 1, *n* = 505; Grade 2, *n* = 1048; Grade 3, *n* = 2128; Grade 4, *n* = 3460. **f** Comparison of mitochondrial state between LGG and HGG by Radar map. **g** Electron microscopic representation of 3 types MRC state in different grade glioma. Green marked Type 1 healthy MRC, blue marked Type 2 unhealthy MRC, and red marked Type 3 damaged MRC. **h** Statistical analysis of 3 types MRC in different grade glioma. Mitochondrial number were: Grade 1, *n* = 72; Grade 2, *n* = 82; Grade 3, *n* = 128; Grade 4, *n* = 270. The data were presented as means ± SD. *P* were calculated by Kruskal-Wallis test (**e**) and ordinary one-way ANOVA (**h**)

We collected glioma tissues from 54 patients who underwent glioma resection at the Department of Neurosurgery, the First Affiliated Hospital of The Fourth Military Medical University between June 2021 and June 2023. The core tissues of the tumors were sampled following Hua Han team's mitochondria electron microscopy sampling and analysis methods [47]. Subsequently, a total of 276 tumor cells with intact cell membrane boundaries were captured under transmission electron microscope, resulting in a dataset comprising 7141 mitochondria (Fig. 1a). According to previous published paper of our laboratory [48], we analyzed the morphological features of mitochondria in different grades of glioma from 4 perspectives: mitochondrial density, mitochondrial size, mitochondrial shape and mitochondrial state (Fig. 1b). To enhance the accuracy and objectivity of our analysis results, we developed a machine learning model for mitochondria identification. The study adopted the two-stage Mask R-CNN algorithm proposed by Kaiming He et al. [32], which exhibits efficiency, objectivity, and accuracy in the field of machine learning for object detection and image segmentation. Additionally, to mitigate over-fitting, the COCO dataset was utilized for model pre-training during mitochondria identification. Thus, we have completed the development of a machine learning model that can accurately identify mitochondria (Fig. 1c). According to the WHO CNS 5 [2], our mitochondria were categorized into four subsets of grade 1, grade 2, grade 3, and grade 4 (Fig. 1d), we found that compared with LGG, the mitochondria of HGG had smaller area, shorter perimeter, higher density, and rounder shape, but there was no significant difference in the area ratio (Fig. 1e). The radar chart summarized the mitochondrial morphological disparities between LGG and HGG (Fig. 1f). To further examine the status of MRC, according to the classification method proposed by Mingqi Han et al., based on the orderliness of MRC observed under the transmission electron microscope [4], glioma mitochondria were categorized into three types in our study: Type 1 exhibited dense and abundant MRC with mostly intact OMM; Type 2 showed sparse MRC (accompanied by occasional incomplete phenomenon in the OMM); and Type 3 displayed severely damaged MRC (often accompanied by incomplete phenomenon in the OMM) (Fig. 1g). The findings indicated that HGG exhibited the highest abundance of healthy Type 1 mitochondria, and the lowest prevalence of less healthy Type 2 and Type 3 mitochondria (Fig. 1h). The current findings suggested a significantly enhanced mitochondrial fission trend in HGG, accompanied by the more structurally complete MRC.

### Bioinformatics analysis and mIHC staining of clinical glioma microarrays screened DRP1 and FIS1 show a trend of high expression with the progression of HGG

**Fig. 2:**
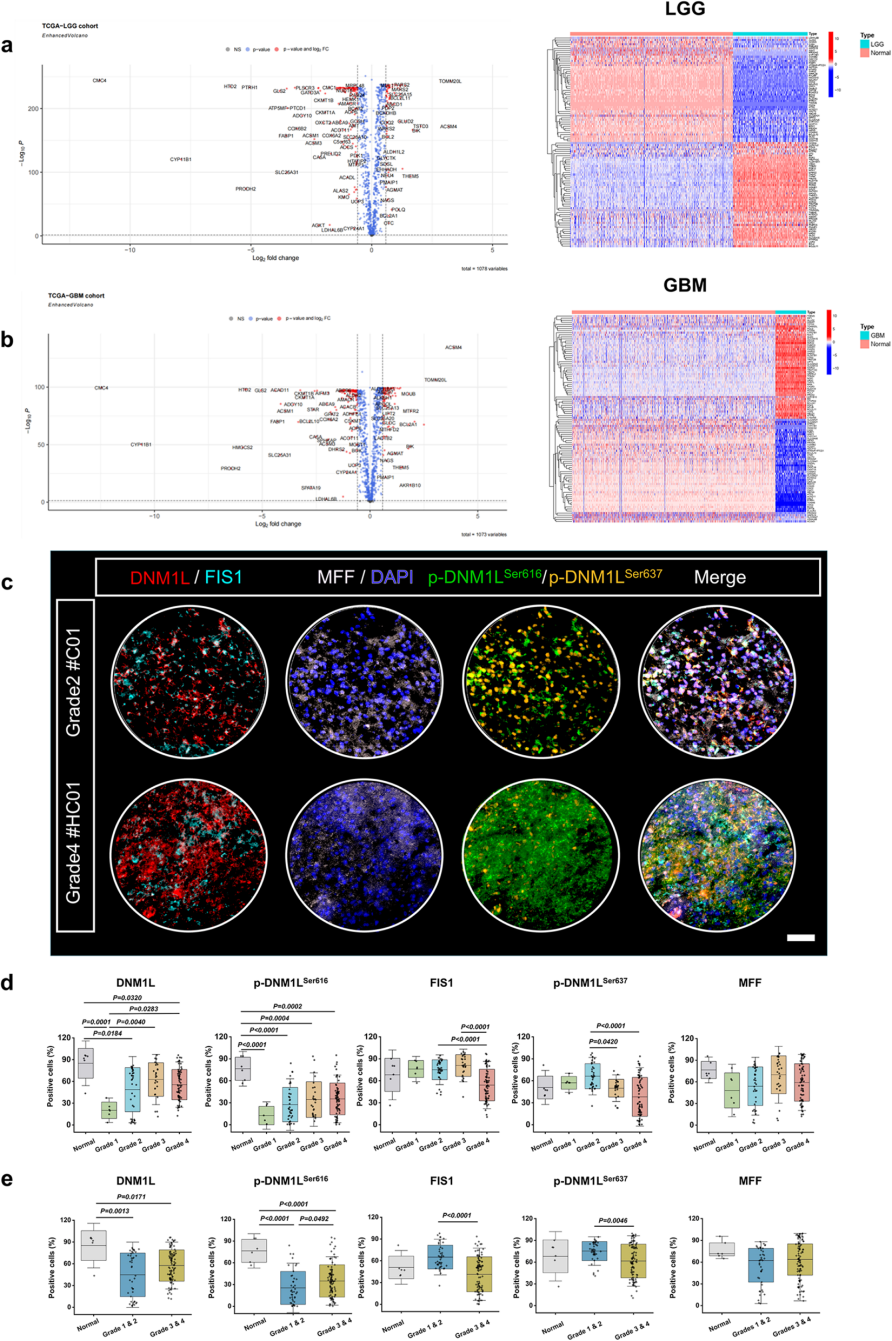
Analyze the differential expression of mitochondria-related genes in human glioma by bioinformatics analysis and mIHC. **a **and** b** The analysis of mitochondria-related genes, down-regulated genes in green and up-regulated genes in red. The heatmap displayed the top 50 MRGs based on their difference multiple. **c** Representative images of microarray staining. Scale bars: 20 µm. **d **and **e** Statistical analysis of the number of DNM1L/DRP1, p-DNM1L^Ser616^, FIS1, p-DNM1L^Ser637^ and MFF positive cells in microarrays (Normal *n* = 6 (*n* = 3, repeated measurement once) Grade 1: *n* = 6; Grade 2: *n* = 37; Grade 3: *n* = 27; Grade 4: *n* = 71). The data were presented as means ± SD. *P* were calculated by Kruskal-Wallis test or ordinary one-way ANOVA (**d** and **e**)

In order to explore the underlying factors contributing to mitochondrial fragmentation and remodeling of MRC in HGG, we then performed bioinformatics analysis on RNA-seq data obtained from LGG, GBM (which was the highest type of HGG), and normal brain tissues using TCGA and GTEx databases (Specific information was provided in Supplementary Table 1). In comparison to normal brain tissues, LGG tissues exhibited down-regulation of 154 MRGs and up-regulation of 84 MRGs (Fig. 2a). Similarly, GBM tissues showed down-regulation of 144 MRGs and up-regulation of 95 MRGs in contrast to normal brain tissues. The heatmap displayed the 50 most significant MRGs based on difference multiple (Fig. 2b). Based on the results of mitochondrial morphological analysis and bioinformatics analysis, we hypothesized that the variations in mitochondria among different grades of glioma are associated with the dynamic regulation of mitochondrial fission and fusion. The mitochondrial fission molecule DNM1L/DRP1 [49], along with its receptors FIS1 [11] and MFF [50], in conjunction with the mitochondrial fusion protein 1 and 2 (MFN1/MFN2) [51], collectively regulate both mitochondrial fission and fusion processes.

Although bioinformatics analysis implied DNM1L/DRP1, FIS1 and MFF as the key modulators for MRC in HGG, it was needed to verify this point in clinical medicine. Hence, we consequently conducted mIHC staining [52] on microarrays comprising tumor tissues from 144 glioma patients and brain tissues from 3 normal human in order to assess the expression levels of DNM1L/DRP1, p-DNM1L^Ser616^ (the positive style of DNM1L/DRP1), p-DNM1L^Ser637^ (the negative style of DNM1L/DRP1), MFF, and FIS1 (Fig. 2c). The sample size of normal human brain tissues was insufficient; thus, we focused our attention on the differential expression of the aforementioned molecules across different grades of glioma. There were no statistically significant differences observed in the expression of MFF among each grade of glioma. However, the levels of DNM1L/DRP1 and p-DNM1L^Ser616^ were significantly elevated in grade 3 and grade 4 glioma compared to grade 1 glioma. Moreover, FIS1 exhibited the highest expression level in grade 3 glioma, while p-DNM1L^Ser637^ displayed the lowest expression level in grade 3 and grade 4 glioma (Fig. 2d). Moreover, the levels of DNM1L/DRP1 and p-DNM1L^Ser616^ were significantly elevated in HGG compared to LGG. Additionally, FIS1 and p-DNM1L^Ser637^ exhibited different trends, but the expression of MFF still did not show any significant disparity between LGG and HGG (Fig. 2e). Based on these findings, we confirmed the aforementioned results in human astrocytes cell lines. The data revealed a significant up-regulation of mitochondrial fission-related molecules in GBM cell lines compared to normal human astrocytes (Supplementary Fig. 2a and b).

These findings suggested that DNM1L/DRP1, FIS1, and MFF may play a crucial role in the occurrence and progression of glioma. To investigate the role of these molecules in HGG mitochondria, we employed lentivirus to modulate the expression of DNM1L/DRP1, FIS1, and MFF in U87MG, U251MG and GSCs. Additionally, considering the inhibitory effect of Mdivi-1 on DNM1L/DRP1 activity [53, 54], we treated U87MG and U251MG with Mdivi-1 for the aforementioned measurements.

### Reducing DNM1L/DRP1 hindered HGG progression, while up-regulating DNM1L/DRP1 could promote the progression of HGG

**Fig. 3:**
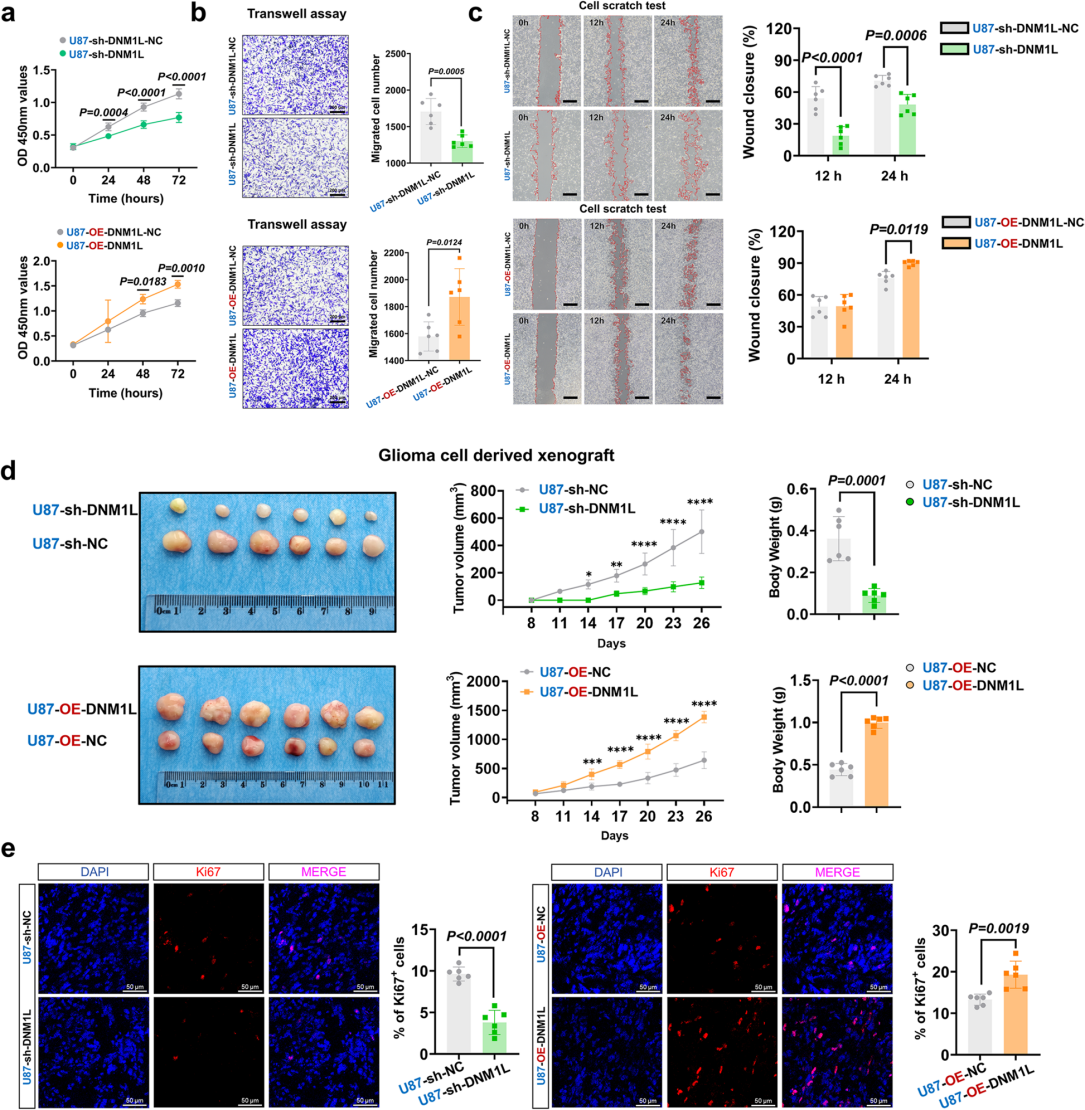
Reducing DNM1L/DRP1 hindered HGG progression, while up-regulating DNM1L/DRP1 can promote the progression of HGG. **a** The CCK-8 assay, detected the proliferation ability of U87MG after regulation of DNM1L/DRP1 (*n* = 6). **b** The Transwell assay, detected the migration ability of U87MG after regulation of DNM1L/DRP1 (*n* = 6). Scale bars: 200 µm. **c** The Wound Healing assay, detected the migration ability of U87MG after regulation of DNM1L/DRP1 (*n* = 6). Scale bars: 500 µm. **d** Glioma cell derived xenograft assay (*n* = 6). *P < 0.05; **P < 0.01; ***P < 0.001; ****P < 0.0001. **e** Representative confocal images of U87MG in sh-DNM1L, sh-DNM1L-NC, OE-DNM1L and OE-DNM1L-NC: DAPI (blue), Ki-67 (red) and merge. Scale bars: 50 µm. Quantitative analysis of % of Ki-67⁺ cells (*n* = 6). The data were presented as means ± SD. *P* were calculated by unpaired two-tailed t-test (**b**,** e**) or two-way ANOVA (**a**,** c**,** d**)

Initially, we focused on modulating the regulation of DNM1L/DRP1 and employed CCK-8, Transwell, and Wound Healing assays to assess the influence of manipulating mitochondrial fission on the progression of HGG. CCK-8 assays demonstrated a significant decrease in cell proliferation rate following sh-DNM1L. Conversely, OE-DNM1L led to a substantial increase in cell proliferation rate (Fig. 3a). The Transwell assays revealed a reduction in migration ability after sh-DNM1L and an increase of approximately 15% upon OE-DNM1L (Fig. 3b). Furthermore, Wound Healing assays indicated a decrease in healing rate after sh-DNM1L and an increase of around 12% following OE-DNM1L (Fig. 3c). More importantly, cell derived xenograft results revealed that U87MG loaded with sh-DNM1L showed significantly decreased tumor volume in a time-dependent manner and tumor weight by 70%, compared with the control group. Conversely, OE-DNM1L showed significantly increased tumor volume in a time-dependent manner and tumor weight, compared with the control group (Fig. 3d). Immunofluorescence analysis of the transplanted tumor cells using Ki-67 revealed that sh-DNM1L significantly suppressed tumor cell proliferation, whereas OE-DNM1L exhibited the opposite effect (Fig. 3e). Similar results were obtained when treating U251MG by the CCK-8 and Wound Healing assay, OE-DNM1L exhibited the similar effect in U251MG by Transwell assay (Supplementary Fig. 3a-c).

The findings of previous studies suggested that the DNM1L/DRP1 inhibitor Mdivi-1 has the potential to induce fusion of mitochondrial networks [55]. The CCK-8, Transwell, and Wound Healing assay collectively demonstrated that Mdivi-1 intervention significantly suppressed the proliferation (Supplementary Fig. 3d), migration (Supplementary Fig. 3e), and healing ability of GBM cells (Supplementary Fig. 3f). These findings suggested that down-regulation of DNM1L/DRP1 restrain both the proliferative capacity and migratory ability of malignant glioma cells.

### The down-regulation of DNM1L/DRP1 would lead to a reduction in FIS1 expression and the inhibition of mitochondrial division, while an increase in the expression of MFF would be observed

**Fig. 4:**
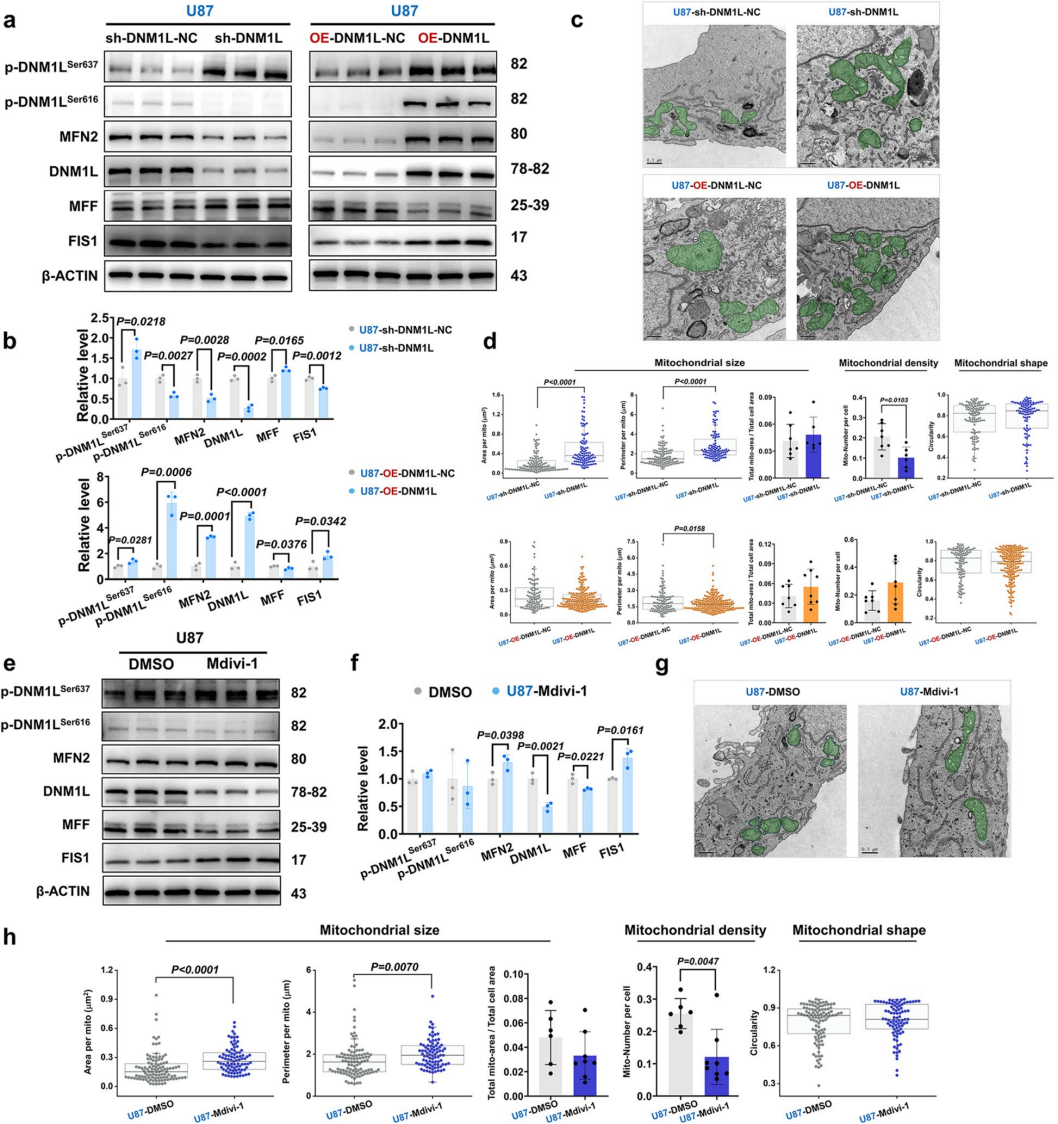
The overexpression of DNM1L/DRP1 significantly increased the expression of FIS1 while suppressing MFF. **a** Western blot, expression level of mitochondria-related proteins in the U87MG when DNM1L/DRP1 was downregulated and overexpressed. **b** Statistical analysis of Western blot after regulation of DNM1L/DRP1. **c** Representative images of electron microscopy after down-regulation and overexpression of DNM1L/DRP1 (U87-sh-DNM1L-NC, *n *= 7; U87-sh-DNM1L, *n* = 6; U87-OE-DNMIL-NC, *n* = 7; U87-OE-DNM1L, *n* = 8). Scale bars: 0.5 µm. **d** Statistical analysis of the mitochondrial size, density and shape after regulation of DNM1L/DRP1 (U87-sh-DNM1L-NC, *n* = 118; U87-sh-DNM1L, *n* = 104; U87-OE-DNMIL-NC, *n* = 108; U87-OE-DNMIL, *n* = 199). **e** Western blot, expressions of mitochondria-related proteins in the U87MG after Mdivi-1 intervention. **f** Statistical analysis of Western blot after Mdivi-1 intervention in U87MG. **g** Representative images of electron microscopy after Mdivi-1 intervention, (U87-DMSO, *n* = 6; U87-Mdivi-1, *n* = 8). Scale bars: 0.5 µm. **h** Statistical analysis of the mitochondrial size, density and shape after Mdivi-1 intervention (U87-DMSO, *n* = 102; U87-Mdivi-1, *n* = 87). The data were presented as means ± SD. *P* were calculated by unpaired two-tailed t-test (**b**,** f**) or Mann-Whitney U test (**d**,** h**)

The previous studies have demonstrated a significant up-regulation of DNM1L/DRP1, FIS1, and MFF in HGG. Consequently, subsequent investigations have focused on modulating the impact of DNM1L/DRP1 on the expression of its downstream molecules, namely FIS1, MFF, and the fusion protein MFN2. The Western blot analysis revealed a significant reduction in the expression level of the target protein in cells transfected with specific shRNA lentivirus (sh-DNM1L) targeting the DNM1L gene, compared to those transfected with a non-specific control lentivirus (sh-DNM1L-NC). Specifically, the average expression level of the control group was normalized to 1, while that of the target protein in the sh-DNM1L group decreased to 0.274, indicating an approximate reduction of 72.6% relative to the control group. Conversely, within the OE-DNM1L-NC group, a significantly lower expression level was observed compared to that in the OE-DNM1L group. After normalizing for lentiviral transfection efficiency, it was found that the expression level of the target protein in the OE-DNM1L group reached 4.9419 times that of the control group, representing an increase of approximately 394.2% over controls. Furthermore, the expressions of MFN2 and FIS1 were reduced, while the expressions of MFF was elevated after sh-DNM1L in U87MG. Conversely, OE-DNM1L administration resulted in an opposite modulation pattern for these molecules (Fig. 4a and b, Supplementary Fig. 3g and h). We noted that OE-DNM1L could increase FIS1 levels in U87MG by 80%, whereas MFF levels were decreased by nearly 20% (Fig. 4a and b), suggesting that FIS1 and MFF have opposite roles in mitochondrial division in GBM. Transmission electron microscope analysis revealed that down-regulation of DNM1L/DRP1 markedly inhibited mitochondrial fission in U87MG, whereas overexpression of DNM1L/DRP1 had no significant effect on mitochondrial fission (Fig. 4c and d).

The Western blot analysis revealed a significant decrease in the expression levels of DNM1L/DRP1 and MFF in GBM cells following treatment with Mdivi-1. Conversely, the expression levels of MFN2 and FIS1 were observed to increase (Fig. 4e and f, Supplementary Fig. 3i and j). Transmission electron microscope further confirmed that Mdivi-1 intervention effectively inhibited mitochondrial division in GBM cells (Fig. 4g and h). These findings indicated that mitochondrial fission may be intricately linked to the biological behavior of gliomas, suggesting that modulation of mitochondrial dynamics could serve as a promising strategy for influencing glioma progression. Nevertheless, to elucidate the specific role and underlying mechanisms of mitochondrial fission in glioma advancement, further comprehensive research and validation are imperative.

### The inhibition of FIS1 attenuated the progression of HGG, whereas the inhibition of MFF enhanced its progression

**Fig. 5:**
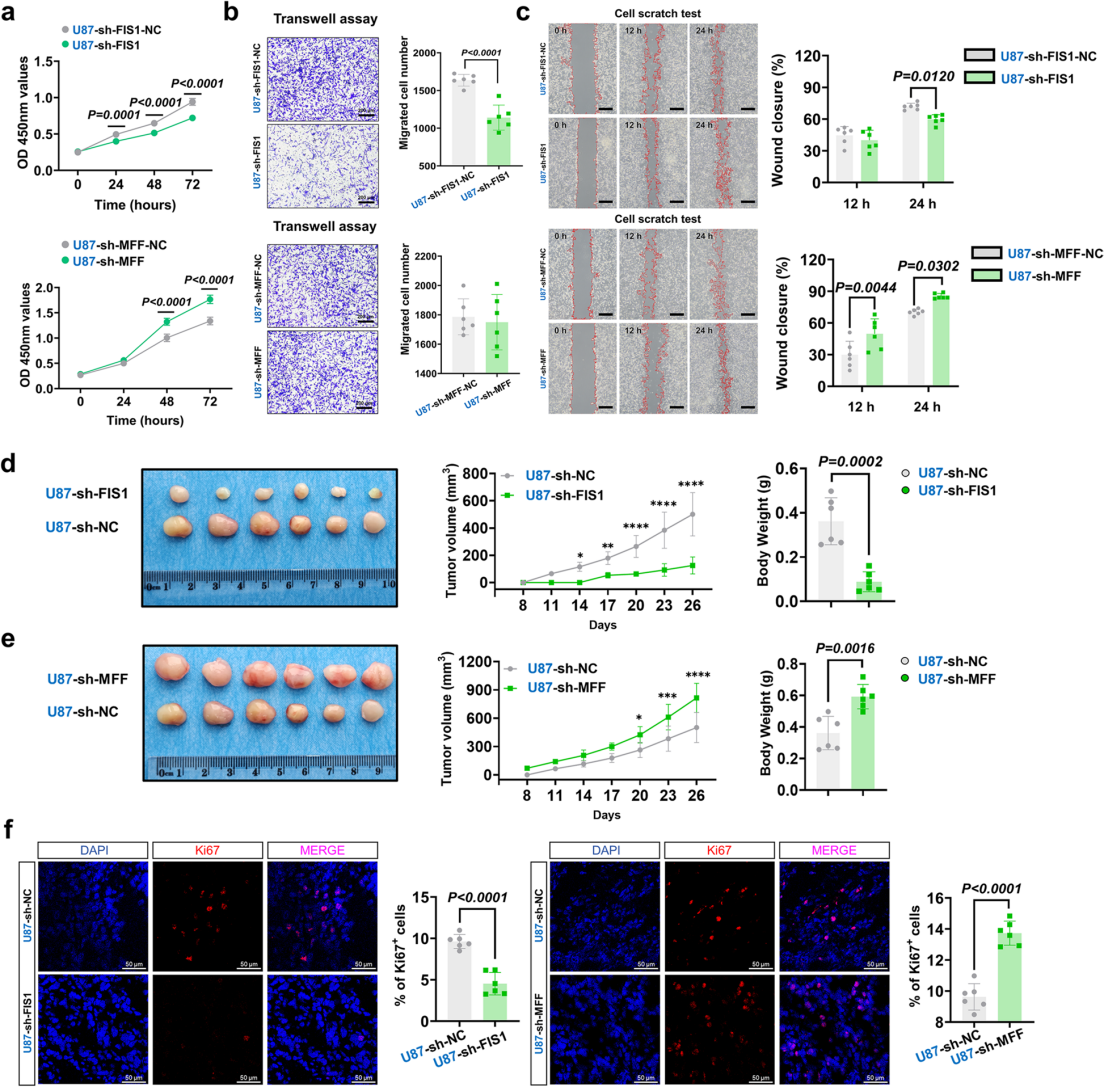
The inhibition of FIS1 attenuated the progression of HGG, whereas MFF played the opposite role. **a** The CCK-8 assay, detected the proliferation ability of U87MG after regulation of sh-FIS1 and sh-MFF (*n* = 6). **b** The Transwell assay, detected the migration ability of U87MG after regulation of sh-FIS1 and sh-MFF (*n* = 6). Scale bars: 200 µm. **c** The Wound Healing assay, detected the migration ability of U87MG after regulation of sh-FIS1 and sh-MFF (*n* = 6). Scale bars: 500 µm. **d** Glioma cell derived xenograft assay after regulation of sh-FIS1 (*n* = 6). *P < 0.05; **P < 0.01; ***P < 0.001; ****P < 0.0001. **e** Glioma cell derived xenograft assay after regulation of sh-MFF (*n* = 6). *P < 0.05; **P < 0.01; ***P < 0.001; ****P < 0.0001. **f** Representative confocal images of U87MG in sh-FIS1, sh-FIS1-NC, sh-MFF and sh-MFF-NC: DAPI (blue), Ki-67 (red) and merge. Scale bars: 50 µm. Quantitative analysis of % of Ki-67⁺ cells (*n* = 6). The data were presented as means ± SD. *P* were calculated by two-way ANOVA (**a**,** c**,** d**,** e**) or unpaired two-tailed t-test (**b**,** f**)

FIS1 and MFF are two receptors of DNM1L/DRP1 [56]. Studies have shown that knockdown of FIS1 can reduce the sensitivity of tumor cells to cisplatin and reduce apoptosis of tumor cells, and FIS1 has the effect of inhibiting cancer [57]. Our third part of the study has revealed that DNM1L/DRP1 plays a positive regulatory role in FIS1, while concurrently exerting negative regulation on its other receptor, MFF. CCK-8 assay revealed a significant decrease in the proliferation rate of U87MG following sh-FIS1, while sh-MFF led to a significant increase in cell proliferation (Fig. 5a). The Transwell assay demonstrated that sh-FIS1 inhibited cell migration in U87MG, whereas sh-MFF showed no significant difference in cell migration (Fig. 5b). Furthermore, sh-FIS1 resulted in a decreased healing rate of U87MG, while sh-MFF increased the healing rate (Fig. 5c). The results were validated through xenograft experiments in mice (Fig. 5d and e). Immunofluorescence analysis of the transplanted tumor cells using Ki-67 revealed that sh-FIS1 significantly suppressed tumor cell proliferation, whereas sh-MFF exhibited the opposite effect (Fig. 5f). Similarly, we performed the same validation on U87MG using OE-FIS1 and OE-MFF, and found that OE-FIS1 promoted tumor progression, while OE-MFF inhibited tumor progression (Supplementary Fig. 4). U251MG related results showed the similar trend except Transwell assay (Supplementary Fig. 5a-l). The aforementioned research suggested that the down-regulation of FIS1 hampers the progression of GBM, whereas MFF exerts an opposing influence.

### The inhibition of FIS1 and MFF impeded mitochondrial fission

**Fig. 6:**
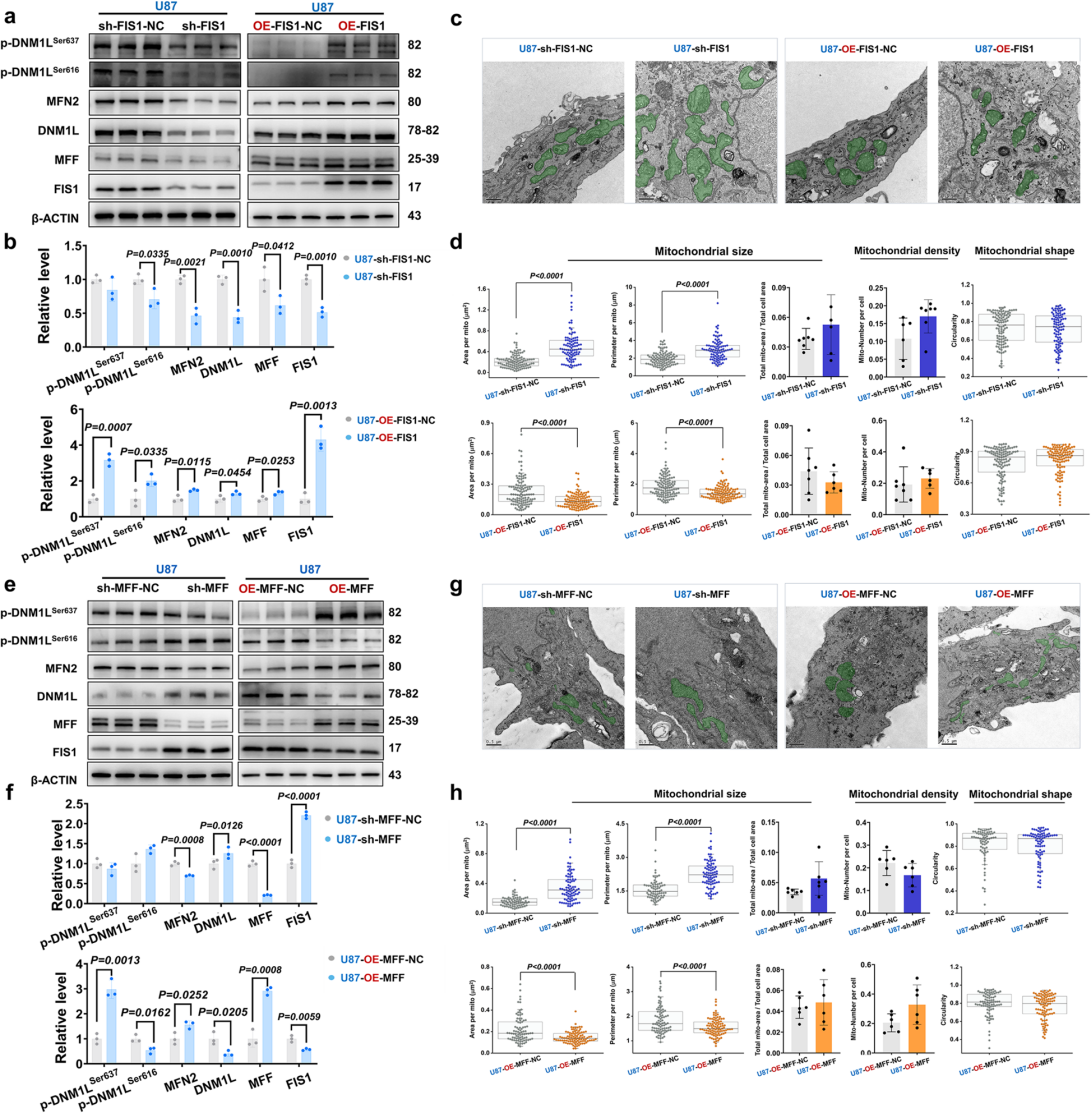
Reducing FIS1 and MFF inhibited mitochondrial division, while the overexpression had the opposite effect. **a** Western blot, expressions of mitochondria-related proteins in the U87MG when FIS1 was down-regulated and overexpressed. **b** Statistical analysis of Western blot after regulation of FIS1. **c** Representative images of electron microscopy after down-regulation and overexpression of FIS1 (U87-sh-FIS1-NC, *n *= 6; U87-sh-FIS1, *n* = 6; U87-OE-FIS1-NC, *n* = 6; U87-OE-FIS1, *n* = 6). Scale bars: 0.5 µm. **d** Statistical analysis of the mitochondrial size, density and shape after regulation of FIS1 (U87-sh-FIS1-NC, *n* = 105; U87-sh-FIS1, *n* = 93; U87-OE-FIS1-NC, *n* = 115; U87-OE-FIS1, *n* = 108). **e** Western blot, expressions of mitochondria-related proteins in the U87MG when MFF was down-regulated and overexpressed. **f** Statistical analysis of Western blot after regulation of MFF. **g** Representative images of electron microscopy after down-regulation and overexpression of MFF (U87-sh-FIS1-NC, *n *= 6; U87-sh-FIS1, *n* = 6; U87-OE-FIS1-NC, *n* = 6; U87-OE-FIS1, *n* = 6). Scale bars: 0.5 µm. **h** Statistical analysis of the mitochondrial size, density and shape after regulation of MFF (U87-sh-MFF-NC, *n* = 72; U87-sh-MFF, *n* = 85; U87-OE-MFF-NC, *n* = 86; U87-OE-MFF, *n* = 98). The data were presented as means ± SD. *P* were calculated by unpaired two-tailed t-test (**b**,** f**) or Mann-Whitney U test (**d**,** h**)

The DNM1L/DRP1-FIS1 axis, being a canonical pathway governing mitochondrial division, was subsequently examined to determine the impact of FIS1 regulation on both mitochondrial division and fusion. The Western blot analysis demonstrated that sh-FIS1 significantly reduced the expression level of FIS1 protein to 0.5161, representing a decrease of 48.39% compared to sh-FIS1-NC. Conversely, OE-FIS1 markedly elevated the expression level of FIS1 protein to 4.3124 times compared to OE-FIS1-NC, indicating an increase of 331.24%. The subsequent analysis revealed that sh-FIS1 led to a down-regulation in the expression of DNM1L/DRP1 and MFF. However, OE-FIS1 yielded contrasting results (Fig. 6a and b, Supplementary Fig. 5m and n). We found sh-FIS1 effectively inhibited mitochondrial fission in U87MG, whereas OE-FIS1 promoted mitochondrial fission (Fig. 6c and d). The aforementioned findings indicated a positive regulatory association between DNM1L/DRP1 and FIS1 in the process of mitochondrial division.

The regulation of DNM1L/DRP1 has been observed to be inversely correlated with the expression trend of MFF in previous studies [58], thus we conducted experiments to validate this finding by manipulating MFF levels in GBM cells. Western blot results confirmed the effect of MFF regulation by down-regulation MFF (sh-MFF) and overexpression MFF (OE-MFF). It should be noted that OE-MFF could decrease expression levels of DNM1L/DRP1 and FIS1, but increased level of p-DNM1L^Ser637^ (Fig. 6e and f, Supplementary Fig. 5o and p). In accordance with the impact of FIS1, sh-MFF also impeded mitochondrial fission, resulting in an enlargement of mitochondria; conversely, OE-MFF facilitated mitochondrial fission (Fig. 6g and h). The above findings suggested that both FIS1 and MFF are capable of inducing mitochondrial fission; however, the distinct modes of fission exert contrasting effects on tumor progression. To elucidate the underlying mechanism responsible for this disparity, we focused our investigation on the impact of different fission modes on mitochondrial function.

### The inhibition of DNM1L/DRP1 and FIS1 leads to suppression of HGG in GSCs

Many types of cancer are believed to be driven by poorly differentiated cell subpopulations, often referred to as cancer stem cells (CSCs). These cells possess the capacity for self-renewal, tumor initiation, and generation of non-tumorigenic progeny. GSCs represent a distinct subpopulation of neural glioma cells with robust self-renewal ability, multi-directional differentiation potential, and resistance mechanisms that contribute significantly to tumor recurrence and poor chemotherapy efficacy [34]. Furthermore, research indicates that GSCs exhibit a preference for OXPHOS energy metabolism over GBM cell lines [35]. Therefore, we conducted repeated interventions and related experiments in GSCs to investigate the impact of DNM1L/DRP1, FIS1 and MFF expression on tumor progression in different metabolic models.

**Fig. 7:**
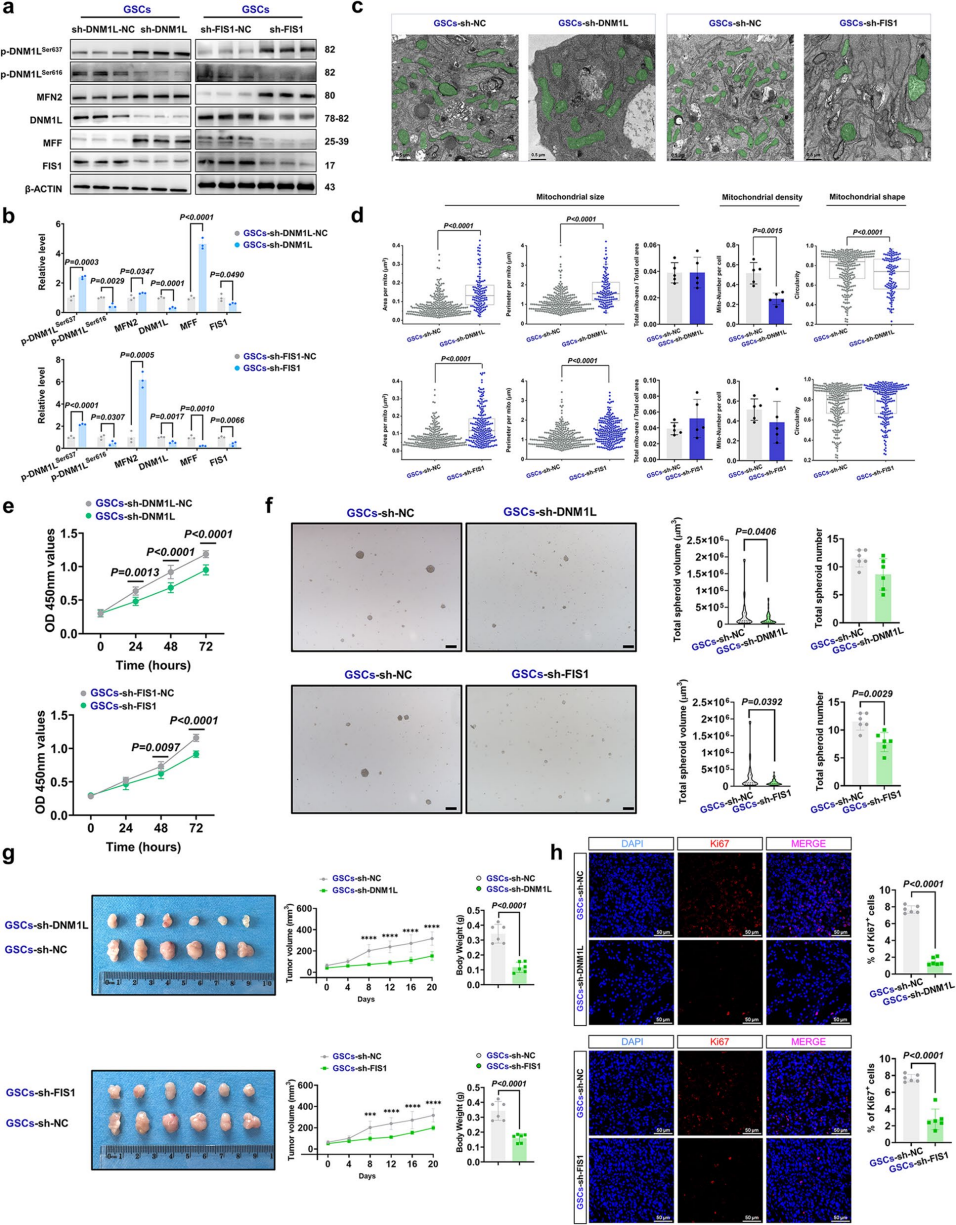
Targeting DNM1L/DRP1-FIS1 axis significantly inhibited OXPHOS in GBM. **a** Western blot, expressions of mitochondria-related proteins in the GSCs when DNM1L/DRP1 or FIS1 was down-regulated. **b** Statistical analysis of Western blot after down-regulation of DNM1L/DRP1 or FIS1. **c** Representative images of electron microscopy after down-regulation of DNM1L/DRP1 or FIS1 (GSCs-sh-DNM1L-NC, *n *= 6; GSCs-sh-DNM1L, *n* = 6; GSCs-sh-FIS1-NC, *n *= 6; GSCs-sh-FIS1, *n* = 6). Scale bars: 0.5 µm. **d** Statistical analysis of the mitochondrial size, density and shape after down-regulation of DNM1L/DRP1 or FIS1 (GSCs-sh-DNM1L-NC, *n *= 272; GSCs-sh-DNM1L, *n *= 119; GSCs-sh-FIS1-NC, *n *= 272; GSCs-sh-FIS1, *n* = 226). **e** The CCK-8 assay, detected the proliferation ability of GSCs after sh-DNM1L and sh-FIS1 (*n* = 6). **f** Representative images and statistical analysis of tumor spheres after down-regulation of DNM1L/DRP1 or FIS1 (GSCs-sh-DNM1L-NC, *n *= 6; GSCs-sh-DNM1L, *n* = 6; GSCs-sh-FIS1-NC, *n *= 6; GSCs-sh-FIS1, *n* = 6). Scale bars: 200 µm. **g** Glioma cell derived xenograft assay after sh-DNM1L or sh-FIS1 (*n* = 6). *P < 0.05; **P < 0.01; ***P < 0.001; ****P < 0.0001. **h **Representative confocal images of GSCs after sh-DNM1L or sh-FIS1: DAPI (blue), Ki-67 (red) and merge. Scale bars: 50 µm. Quantitative analysis of % of Ki-67⁺ cells (*n* = 6). The data were presented as means ± SD. *P* were calculated by unpaired two-tailed t-test (**b**,** h**), Mann-Whitney U test (**d**) or two-way ANOVA (**e**,** g**)

Initially, we utilized the crucial molecular markers CD133 and SOX2 for fluorescent labeling and identification of GSCs (Supplementary Fig. 6) [36]. In GSCs, both sh-DNM1L and sh-FIS1 exhibited similar effects on mitochondrial fission-related molecules as observed in U87MG and U251MG (Fig. 7a-d). Furthermore, sh-DNM1L and sh-FIS1 significantly impeded the proliferation of GSCs (Fig. 7e), as well as reduced the number and volume of tumor spheres (Fig. 7f), along with diminishing the weight and size of heterotopic tumors (Fig. 7g). Immunofluorescence analysis of Ki-67 on the heterotopic xenografts demonstrated that sh-DNM1L and sh-FIS1 notably suppressed GSCs cell proliferation (Fig. 7h). Conversely, OE-DNM1L and OE-FIS1 markedly facilitated GSCs progression (Supplementary Fig. 7a-h). Similarly, MFF expression mirrored that in U87MG and U251MG, exhibiting a diametrically opposite pattern to DNM1L/DRP1-FIS1 expression levels (Supplementary Fig. 8a-h).

### The inhibition of DNM1L/DRP1 and FIS1 significantly attenuated the basal respiration and ATP production in HGG mitochondria

**Fig. 8:**
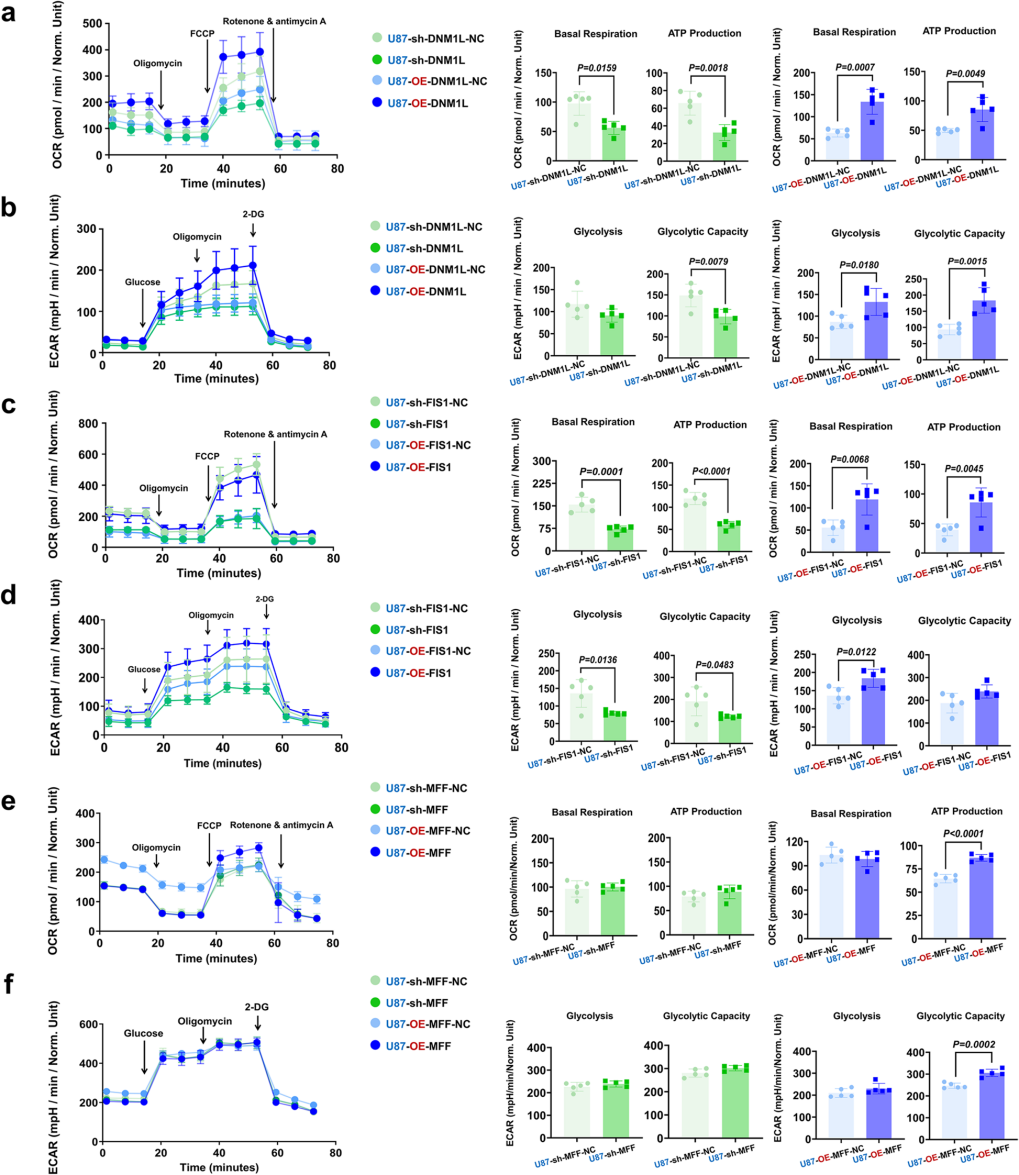
Targeting DNM1L/DRP1-FIS1 axis significantly inhibited OXPHOS in GBM, while up-regulating the axis had opposite effect. **a** Seahorse XF Cell Mitochondria Stress Test, statistical analysis of various indices representing the mitochondrial OXPHOS ability after regulation of DNM1L/DRP1 (*n* = 5). **b** Seahorse XF Glycolysis Rate Assay, statistical analysis of various indices representing the mitochondrial glycolysis ability after regulation of DNM1L/DRP1 (*n* = 5). **c** Seahorse XF Cell Mitochondria Stress Test, statistical analysis of various indices representing the mitochondrial OXPHOS ability after regulation of FIS1 (*n* = 5). **d** Seahorse XF Glycolysis Rate Assay, statistical analysis of various indices representing the mitochondrial glycolysis ability after regulation of FIS1 (*n* = 5). **e** Seahorse XF Cell Mitochondria Stress Test, statistical analysis of various indices representing the mitochondrial OXPHOS ability after regulation of MFF (*n* = 5). **f** Seahorse XF Glycolysis Rate Assay, statistical analysis of various indices representing the mitochondrial glycolysis ability after regulation of MFF (*n* = 5). The data were presented as means ± SD. *P* were calculated by unpaired two-tailed t-test

The ability of tissue invasion and metastasis is a fundamental characteristic of malignant tumors, closely associated with the formation of pseudopodia. This process entails dynamic alterations in microfilaments and microtubules, necessitating a substantial amount of ATP [59, 60]. Studies have shown that OXPHOS is essential for tumor cell proliferation, and mitochondrial oxidative phosphorylation plays a very important role in the occurrence and development of tumors [18, 61]. We found that OCR and ECAR of glioma cells were significantly increased compared with normal human astrocytes (Supplementary Fig. 9a and b). Further research revealed that targeting sh-DNM1L significantly decreased basal respiration rates and ATP production by at least 50% in OCR analysis compared to sh-DNM1L-NC, implying the presence of the effect of DNM1L/DRP1 on mitochondrial internal function. Conversely, OE-DNM1L markedly increased basal respiration and ATP generation by at least 40% compared to OE-DNM1L-NC (Fig. 8a). We got the similar results in U251MG (Supplementary Fig. 9c). Considering the high glycolysis state was the feature of HGG, we then studied the effect of DNM1L/DRP1 on glycolysis. ECAR analysis revealed that sh-DNM1L significantly reduced glycolysis capacity by 60% in U87MG and reduced glycolysis by 50% in U251MG. OE-DNM1L significantly increased glycolysis and glycolysis capacity by at least 50% (Fig. 8b, Supplementary Fig. 9d), providing evidence for DNM1L/DRP1 participation in HGG. The OCR detection revealed that the sh-FIS1 resulted in the inhibition of mitochondrial respiratory function, while OE-FIS1 significantly enhanced this function (Fig. 8c). The ECAR detection demonstrated that sh-FIS1 markedly reduced glycolysis function, whereas its overexpression enhanced glycolysis (Fig. 8d). U251MG related results showed the similar trend (Supplementary Fig. 9e and f).

The OCR experiment demonstrated no alterations in oxygen consumption and ATP production of GBM cells following sh-MFF (Fig. 8e). Similarly, the ECAR experiment revealed no significant changes in glycolysis after sh-MFF, whereas OE-MFF promoted glycolytic capacity (Fig. 8f). In U251MG, the OCR experiment demonstrated that sh-MFF did not alter basal respiration and ATP production; however, OE-MFF led to a reduction in basal respiration with an increase in ATP production. Furthermore, the ECAR experiment showed that sh-MFF had no significant effect on glycolysis ability, while OE-MFF resulted in an increased glycolysis but a decrease in glycolytic capacity. These findings suggested that overexpression of MFF may trigger a metabolic compensation mechanism in U251MG leading to decoupling of glycolytic efficiency and rate (Supplementary Fig. 9g and h). Similarly, when we targeted the regulation of DNM1L/DRP1 and FIS1 in GSCs, the observed changes in OCR followed a similar trend to that of U87MG and U251MG, while the changes in ECAR exhibited a significantly opposite trend. The impact of sh-MFF on ECAR resembles the outcomes observed when DNM1L/DRP1 and FIS1 are downregulated. This observation demonstrates that, compared to U87MG/U251MG, GSCs exhibiting OXPHOS as their primary metabolic pattern repressed glycolysis function and displayed increased reliance on OXPHOS following substantial augmentation of mitochondrial fission mediated by DNM1L/DRP1-FIS1 (Supplementary Fig. 10a).

The OCR/ECAR, an indicator of tumor cell preference for glycolysis [62], increased following DNM1L up-regulation (Supplementary Fig. 11a and b) and decreased upon sh-FIS1 (Supplementary Fig. 11c and d). Glioma cells tended to oxygenate more after OE-MFF (Supplementary Fig. 11e and f).

The aforementioned findings indicated that DNM1L/DRP1 might interact with its receptor FIS1 and facilitate the progression of HGG by augmenting OXPHOS.

### The inhibition of DNM1L/DRP1 and FIS1 resulted in a significant reduction in MMP and ROS levels, leading to the disruption of the MRC structure

The stability of mitochondrial MMP, which is crucial for promoting the production of OXPHOS and ATP in cells, thereby maintaining the normal physiological function of cells. Enhanced mitochondrial function is often accompanied by increased levels of MMP. ROS, as a byproduct of mitochondrial OXPHOS, reflects the state of mitochondrial function [63]. The literatures have documented defects in mitochondrial function, dynamics, and quality control in numerous tumor-related studies, characterized by a significant disruption of MMP and ROS levels, as well as dysregulated bioenergy and metabolism [16, 64, 65]. The levels of ROS, primarily generated by mitochondria, are typically elevated in cancer cells [66]. The levels of ROS in glioma cells were found to be significantly higher compared to those in normal human astrocytes. In comparison to normal human astrocytes, we observed that the MMP levels in the U87MG did not exhibit a significant decrease, whereas the U251MG demonstrated a notable reduction in MMP expression (*p* = 0.0435). This observation indicated potential disparities in MMP expression among different glioma cell lines, which could be attributed to their distinct genetic backgrounds, biological characteristics, and adaptability to the tumor microenvironment (Supplementary Fig. 12a and b).

**Fig. 9:**
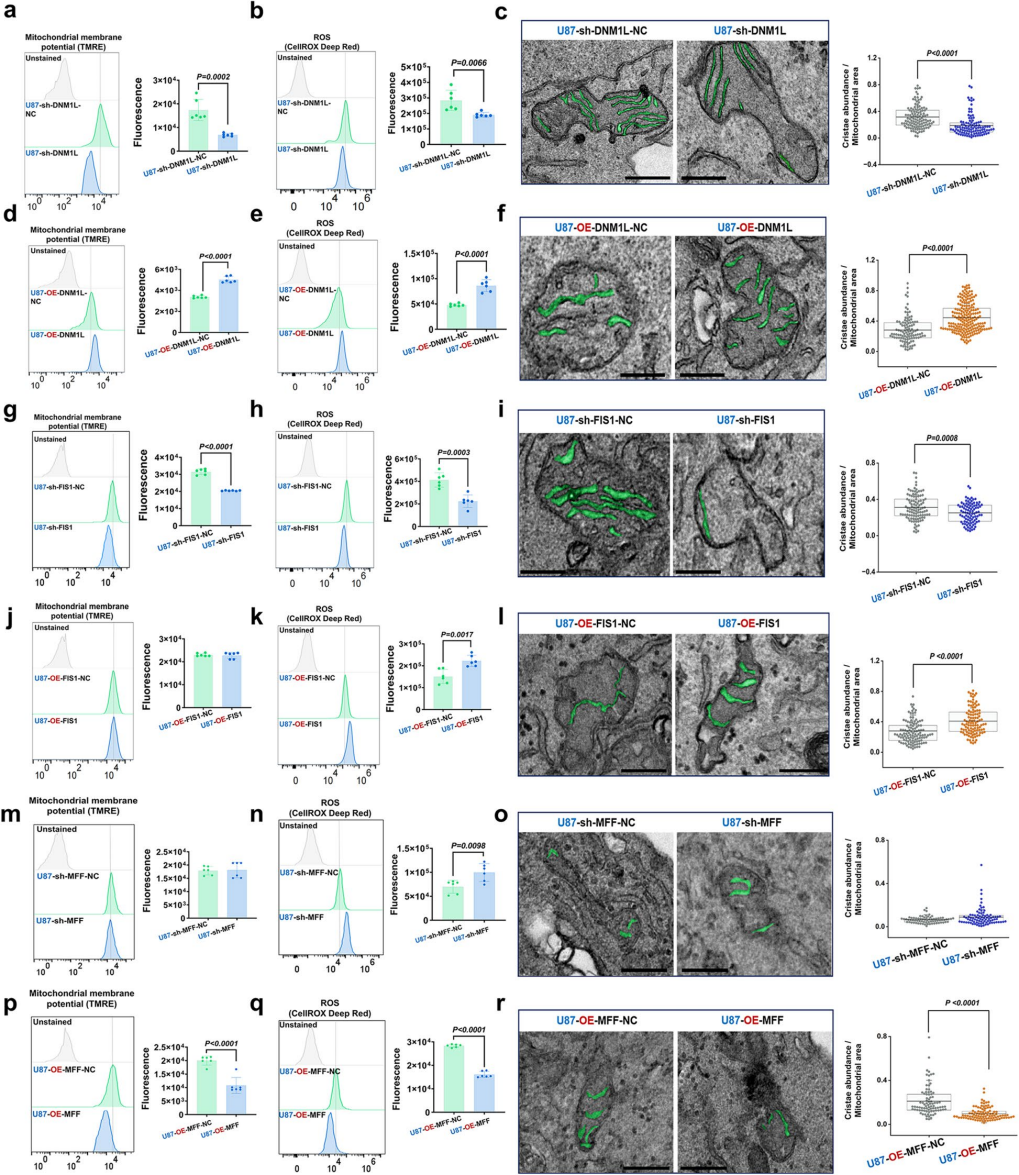
The inhibition of DNM1L/DRP1-FIS1 axis impeded MRC structure and damaged mitochondrial quality. **a **and** b** Detected the MMP and ROS of U87MG after down-regulation of DNM1L/DRP1, the mean fluorescence intensity of positive cells was measured. (*n* = 6). **c** The cross-section of the MRC was highlighted in green in the electron microscope images, and a statistical analysis was conducted to determine the extent of MRC occupancy. Scale bars: 0.3 µm. **d **and** e** Detected the MMP and ROS of U87MG after overexpression of DNM1L/DRP1, the mean fluorescence intensity of positive cells was measured (*n* = 6). **f** The details depicted in the picture align with those presented in (**c**). **g **and** h** Detected the MMP and ROS of U87MG after down-regulation of FIS1, the mean fluorescence intensity of positive cells was measured (*n* = 6). **i** The details depicted in the picture align with those presented in (**c**). **j **and** k** Detected the MMP and ROS of U87MG after overexpression of FIS1, the mean fluorescence intensity of positive cells was measured (*n* = 6). **l** The details depicted in the picture align with those presented in (**c**). **m **and** n** Detected the MMP and ROS of U87MG after down-regulation of MFF, the mean fluorescence intensity of positive cells was measured. (*n* = 6). **o** The details depicted in the picture align with those presented in (**c**). **p **and** q** Detected the MMP and ROS of U87MG after overexpression of MFF, the mean fluorescence intensity of positive cells was measured (*n* = 6). **r** The details depicted in the picture align with those presented in (**c**). The data were presented as means ± SD. *P* were calculated by unpaired two-tailed t-test (**a**,** b**,** d**,** e**,** g**,** h**,** j**,** k**,** m**,** n**,** p**,** q**) or Mann-Whitney U test (**c**,** f**,** i**,** l**,** o**,** r**)

Further considering MMP and ROS could reflect MRC state [16], we performed flow cytometry analysis to detect MMP level and ROS level in U87MG. In addition, mitochondrial OXPHOS occurs primarily in the mitochondrial MRC [67, 68]. More importantly, we next quantified the abundance of MRC by calculating the ratio of respiratory cristae area to mitochondrial area. It was shown that sh-DNM1L significantly damaged MMP and ROS levels, and abundance of MRC compared to sh-DNM1L-NC (Fig. 9a-c, Supplementary Fig. 12c and d), which was consistent with results of OXPHOS and HGG progression. On the contrary, OE-DNM1L significantly stabilized MMP and ROS levels, and abundance of MRC (Fig. 9d-f, Supplementary Fig. 12e and f). Treatment of sh-FIS1 damaged ROS and abundance of MRC, and resulted in a decrease in MMP level (Fig. 9g-i, Supplementary Fig. 12g and h), while OE-FIS1 stabilized ROS and abundance of MRC, but have no effect on MMP (Fig. 9j-l, Supplementary Fig. 12i and j). In addition, sh-MFF significantly increased the content of ROS in glioma cells, but had no effect on the remodeling of MMP and MRC (Fig. 9m-o, Supplementary Fig. 12k and l); while OE-MFF significantly decreased MMP and ROS, and the abundance of MRC was also reduced (Fig. 9p-r, Supplementary Fig. 12m and n). Previous studies have demonstrated that the mitochondrial fission inhibitor Mdivi-1 effectively suppresses the proliferation of malignant cells [53]. In this study, U87MG and U251MG were treated with a concentration of 25 µmol/L Mdivi-1. It was shown that Mdivi-1 significantly damaged ROS and abundance of MRC, but did not change MMP (Supplementary Fig. 12o-t). Through these experiments, we could conclude that the DRP1/FIS1 axis play a crucial role in regulating mitochondrial quality in GBM by reshaping the MRC. Similarly, we observed consistent patterns when targeting the GSCs' DNM1L/DRP1, FIS1, and MFF (Supplementary Fig. 13a-r).

### Repression of DNM1L/DRP1 and FIS1 significantly inhibited mitophagy

Previous studies have shown that MRC could be affected by mitochondrial quality control system, including mitophagy and anti-oxidative stress. ROS, as a byproduct of OXPHOS, is significantly increased in tumor cells [66]. The copper-zinc superoxide dismutase 1 (SOD1) is a ubiquitously expressed cytoplasmic antioxidant enzyme, and its high expression in cancer cells maintains an elevated level of ROS homeostasis [69, 70]. Inhibiting SOD1 activity in cancer cells can effectively modulate the ROS signaling pathway, impede the growth and proliferation of cancer cells, arrest the cell cycle progression, and promote apoptosis in cancer cells [71]. Increased mitochondrial fission promotes the production of ROS in the cytoplasm, thereby upregulating the expression of DNM1L/DRP1 and FIS1 and promoting the occurrence and development of tumors [72].

The process of mitochondrial division is intricately linked to its quality control, with mitophagy serving as the primary modality for regulating mitochondrial quality. Mitophagy is a highly conserved cellular process in eukaryotic cells that selectively eliminates dysfunctional or superabundant mitochondria through an autophagic mechanism. It is crucial for maintaining the stability of cellular energy metabolism and intracellular homeostasis [73]. Autophagy-related protein 5 (ATG5) plays a pivotal role in autophagosome formation, being involved in the generation and expansion of autophagosome precursors. ATG5 contributes to the assembly of the ATG5-ATG12-ATG16L complex, which localizes to the membrane of autophagosome precursors and interacts with microtubule-associated protein 1 light chain 3 (LC3) to facilitate expansion and closure of the autophagosome membrane [74]. LC3 serves as a widely utilized marker for autophagy, participating in autophagosome formation as well as serving as a structural component. During autophagy, activation by ATG7 (E1-like enzyme) and ATG3 (E2-like enzyme) leads to covalent binding of LC3-I to phosphatidylethanolamine (PE), forming the membrane-bound form LC3-II. Subsequently, LC3-II rapidly translocates to nascent autophagosomes where it associates with them. An increase in LC3-II levels typically indicates heightened levels of autophagy activity [75]. p62 (also known as SQSTM1) functions as a selective receptor for autophagy. During formation of an autophagosome, p62 acts as an intermediary between LC3 and polyubiquitinated proteins before being selectively sequestered into these structures for subsequent degradation by proteolytic enzymes within lysosomes [76]. PINK1, a serine/threonine kinase located on depolarized mitochondria, represents a key initiator molecule for ubiquitin-dependent mitophagy induction. Upon mitochondrial damage, PINK1 accumulates at OMM where it becomes activated; subsequently recruiting PARKIN, leading to ubiquitination of OMM proteins thereby marking them for recognition and degradation by autophagosomes [77]. Furthermore, hypoxia induces mitophagy via anon-ubiquitin-dependent pathway. The receptor of Nip-like protein X (NIX) and FUN14 domain containing 1 (FUNDC1) on the OMM can directly bind to LC3 without ubiquitination, thereby initiating mitophagy [78].

**Fig. 10:**
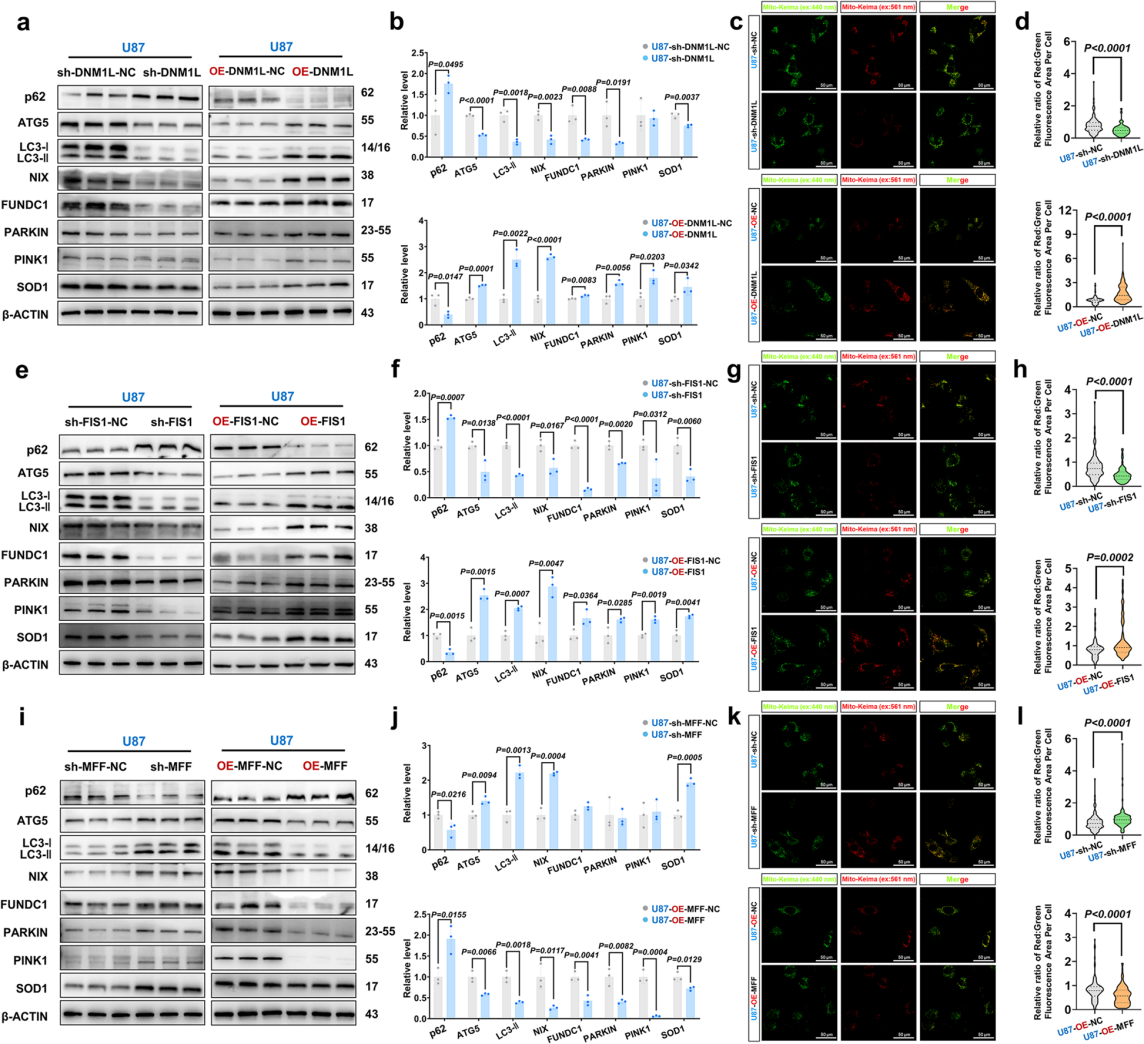
Mitophagy and anti-oxidant stress in glioma were mainly related to DNM1L/DRP1-FIS1 axis. **a** Western blot, expressions of mitochondria-related proteins in the U87MG when DNM1L/DRP1 was downregulated and overexpressed. **b** Statistical analysis of Western blot after regulation of DNM1L/DRP1. **c **Representative confocal images of U87MG after regulation of DNM1L/DRP1: Mito-Keima 440 nm (green), Mito-Keima 561 nm (red) and merge. Scale bars: 50 µm. **d** Statistical analysis of of red/green (OE-DNM1L: *n* = 135; sh-DNM1L: *n* = 125; OE-DNM1L-NC: *n* = 117; sh-DNM1L-NC: *n* = 130). **e** Western blot, expressions of mitochondria-related proteins in the U87MG when FIS1 was down-regulated and overexpressed. **f** Statistical analysis of Western blot after regulation of FIS1. **g **Representative confocal images of U87MG after regulation of FIS1: Mito-Keima 440 nm (green), Mito-Keima 561 nm (red) and merge. Scale bars: 50 µm. **h** Statistical analysis of of red/green (OE-FIS1: *n* = 123; sh-FIS1: *n* = 138; OE-FIS1-NC: *n* = 117; sh-FIS1-NC: *n* = 130). **i** Western blot, expressions of mitochondria-related proteins in the U87MG when MFF was down-regulated and overexpressed. **j** Statistical analysis of Western blot after regulation of MFF. **k **Representative confocal images of U87MG after regulation of MFF: Mito-Keima 440 nm (green), Mito-Keima 561 nm (red) and merge. Scale bars: 50 µm. **l** Statistical analysis of of red/green (OE-MFF: *n* = 119; sh-MFF: *n* = 124; OE-MFF-NC: *n* = 117; sh-MFF-NC: *n* = 130). The data were presented as means ± SD. *P* were calculated by unpaired two-tailed t-test (**b**, **f**, **j**) or Mann-Whitney U test (**d**, **h**, **l**)

Study demonstrated a close correlation between changes in mitochondrial ROS and MMP with PINK1 and PARKIN [66, 79]. In the course of investigating the autophagy mechanism in glioma cells, Western blot analysis unveiled a pivotal finding: sh-DNM1L treatment led to a significant down-regulation of ATG5 and LC3-II protein expression in glioma cells, concurrently accompanied by a pronounced increase in p62 protein levels. This expression profile strongly indicated that autophagic flux was markedly inhibited, as p62 accumulation was frequently regarded as an indicator of compromised autophagic degradation. Further examination of the mitophagy pathway revealed that both ubiquitin-dependent markers PINK1 and PARKIN, along with non-ubiquitin-dependent markers NIX and FUNDC1, were significantly suppressed by sh-DNM1L treatment. Additionally, the expression of the antioxidant enzyme SOD1 was also notably down-regulated. In contrast, when glioma cells were subjected to OE-DNM1L treatment, these molecular expression changes exhibited an opposing trend, suggesting that DNM1L played a crucial role in modulating cellular autophagy and mitophagy (Fig. 10a-b; Supplementary Fig. 14a-b). Furthermore, the Mito-Keima adenovirus technology was employed to monitor the occurrence and progression of mitophagy by analyzing variations in fluorescence signals. Under neutral pH conditions, Mito-Keima emits green fluorescence upon excitation at 440 nm; conversely, under acidic pH conditions, it emits red fluorescence when excited at 561 nm. The alteration in the ratio of fluorescence signals (561 nm/440 nm) serves as a direct indicator of the dynamic changes occurring in mitophagy. The findings indicated that sh-DNM1L markedly diminished mitophagy levels, whereas OE-DNM1L significantly augmented mitophagy (Fig. 10c-d, Supplementary Fig. 14c-d). Similarly, sh-FIS1 exhibited pronounced inhibitory effects on both mitophagy levels and SOD1 expression in glioma cells; conversely, OE-FIS1 elicited comparable positive regulatory effects as OE-DNM1L. This suggested a potential synergistic role of FIS1 with DNM1L in modulating mitophagy (Fig. 10e-h, Supplementary Fig. 14e-h). Interestingly, OE-MFF significantly down-regulated the expression of ATG5, LC3-II, and various mitophagy-related markers (including PINK1, PARKIN, NIX, and FUNDC1), which was accompanied by an accumulation of p62 and a reduction in SOD1 expression in U87MG. This indicated that both mitophagy flux and activity were markedly inhibited. In comparison to OE-DNM1L and OE-FIS1, the impact of OE-MFF on mitophagy levels and SOD1 expression in glioma cells exhibited notable differences, further underscoring the distinct roles played by different molecules in modulating mitophagy and cellular oxidative states within glioma cells (Fig. 10i-l; Supplementary Fig. 14i-l). Such distinctions enhanced our comprehension of the intricate balance between mitochondrial dynamics and autophagy regulatory mechanisms, also motivated us to delve deeper into the complex network of interactions among these molecules and their collective influence on the biological behavior of glioma cells. These findings held significant implications for elucidating the pathogenesis of glioma as well as identifying novel therapeutic targets. Subsequently, we validated the regulatory functions of DNM1L/DRP1, FIS1, and MFF in autophagic flux and mitophagy within GSCs by conducting analogous treatment experiments, yielding results consistent with those observed in glioma cell lines (Supplementary Fig. 15a-l). This finding reinforced our previous observations in glioma cells and elucidated the roles of these molecules within GSCs, a subpopulation exhibiting characteristics akin to tumor stem cells. These findings were subsequently validated in GSCs, confirming that MRC remodeling in GBM cells is closely associated with the expression of the DNM1L/DRP1-FIS1 axis. During this process, HGG up-regulated the DNM1L/DRP1-FIS1 axis and enhanced ubiquitin-dependent mitophagy and non-ubiquitin-dependent mitophagy, thereby significantly enhancing MRC stability. Moreover, it substantially fortified HGG cells' anti-oxidative stress. Conversely, MFF exerts an opposing effect.

**Fig. 11:**
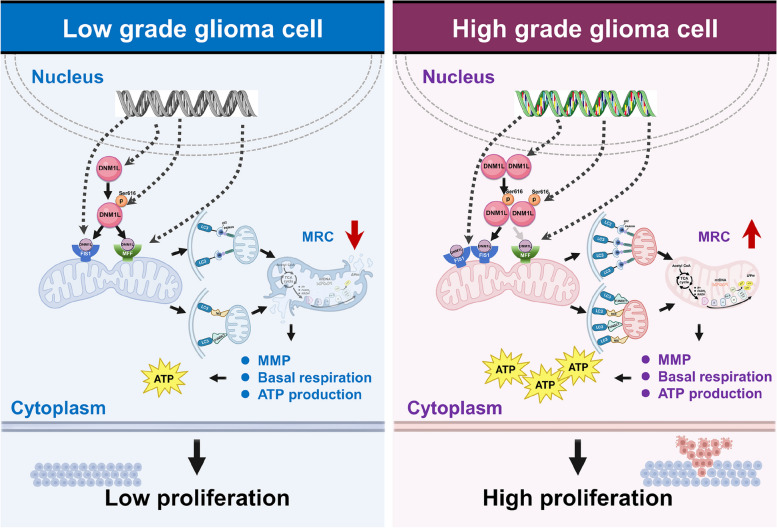
Graphical abstract. Compared to LGG, HGG exhibited elevated levels of DNM1L/DRP1 and its receptor FIS1, demonstrating a more pronounced inclination towards mitochondrial division. Additionally, it displayed significantly enhanced autophagy and antioxidant stress resistance, leading to structural remodeling of the MRC and augmentation of OXPHOS, ultimately conferring robust progression capabilities. Conversely, another DNM1L/DRP1 receptor MFF played an opposing role in the advancement of MRC, OXPHOS, and HGG. Therefore, we propose that targeted down-regulation of the DNM1L/DRP1-FIS1 axis holds potential for therapeutic intervention in HGG

In conclusion, targeted down-regulation of the DNM1L/DRP1-FIS1 axis in glioma can regulate mitophagy and impact the remodeling and OXPHOS function of MRC, ultimately inhibiting tumor occurrence and progression (Fig. 11).

## Discussion

It has been shown that dynamic of MRC and its OXPHOS outcome drive HGG progression, however, due to the deficiency of its underlying mechanism, clinical medicine had to face the severe situation that the patients with GBM, the highest type of HGG, only have survival duration no more than 2 years [3]. This study revealed distinct roles of mitochondrial fission-related proteins in tumor development. The DNM1L/DRP1-FIS1 axis enhanced the stability of MRC in HGG through autophagy, thereby reinforcing its OXPHOS and antioxidant stress resistance, ultimately facilitating robust progression of HGG. Conversely, elevated expression of MFF impeded tumor growth. Consequently, down-regulating the DNM1L/DRP1-FIS1 axis held potential for HGG treatment.

We propose that changes in the glioma microenvironment trigger mitochondrial stress, which is manifested as dramatic changes in proteins involved in mitochondrial dynamics as well as changes in mitochondrial structure. In LGG, the expression of DNM1L/DRP1-FIS1 axis is decreased, while the expression of MFF is increased, resulting in abnormal mitochondrial dynamics, which damages the stability of the inner mitochondrial membrane (IMM) structure, causes the disorder of the respiratory cristae structure composed of the IMM, causes the damage of the electron transport chain of the respiratory cristae, and finally leads to the mitochondria-sourced energy crisis. At this time, LGG tends to glycolysis energy, therefore, clinical reducing energy supply can effectively attack LGG, and clinically achieve the effect of prolonged survival of patients with LGG after treatment. Unfortunately, in contrast to LGG, HGG show a surprising adaptability in gene regulation, as shown by increased expression of DNM1L/DRP1-FIS1 and decreased expression of MFF. The changes in mitochondrial dynamics ensure the OMM of HGG to be highly plastic, resistant and elastic. The stability of the OMM further protects the structural and functional stability of the IMM. It is characterized by the dense structure of the respiratory ridge on the IMM and the enhanced effect of electron transport and OXPHOS, which turns the mitochondria-derived energy crisis into the activation of the dual energy supply systems of mitochondria and glycolysis. At this time, HGG is no longer trapped in the unfavorable microenvironment and even the operation cutting off energy supply. As a result, clinically patients with HGG will rapidly undergo the decline of healthy cells after any current treatment, but the fate of glioma cell growth cannot be reversed. Our study for the first time suggested that mitochondrial dynamic-related protein is an important mechanism for the development of HGG by stabilizing the structure and function of MRC, and blocking the mitochondrial dynamic-related protein DNM1L/DRP1-FIS1 axis can treat the further progression of HGG by disrupting the structure and function of MRC. More importantly, previous studies have reported a close association between mitochondrial fission and tumor progression. Furthermore, our findings indicated that the proteins involved in mitochondrial fission play contrasting roles in glioma development. Specifically, targeted inhibition of the DNM1L/DRP1-FIS1 axis demonstrated significant anti-tumor effects, whereas inhibiting MFF expression exhibited notable pro-tumor effects. These results provided compelling evidence for the clinical application of targeting mitochondrial fission to treat glioma.

Mitochondria are organelles in cells that are responsible for important life activities such as ATP synthesis, fatty acid β-oxidation, tricarboxylic acid (TCA) cycle, REDOX homeostasis, and respiratory chain [80]. In cancer, mitochondria play a crucial role. Firstly, mitochondria play a key role in the metabolism and energy production of cancer cells. Cancer cells often have abnormal metabolic properties, such as the Warburg effect, and a preference for glycolysis to produce energy even under aerobic conditions. This altered mode of metabolism is associated with defects in the mitochondrial respiratory chain, allowing cancer cells to utilize glucose for anaerobic glycolysis to produce lactate and ATP. Secondly, mitochondria are also involved in cellular activities such as signal transmission between cells, differentiation, and life cycle. Dysfunction of mitochondria may cause cells to initiate apoptotic programs, and cells that "attempt suicide" may become cancer cells. Therefore, mitochondrial dysfunction is closely related to cancer initiation and progression. In addition, mitochondria contain genes encoding enzymes associated with different malignancies. The expression and regulation of these genes play an important role in the initiation and progression of cancer. Therefore, mutations and aberrant expression of the mitochondrial genome are also an important factor in cancer development. Finally, mitochondria also hold important promise in cancer therapy. Some drugs and treatments targeting mitochondria are under investigation, such as mitochondria-targeted anticancer drugs and photodynamic therapy. These therapies aim to treat cancer by directly acting on mitochondria and disrupting the energy metabolism and survival mechanisms of cancer cells. In conclusion, there is a close relationship between cancer and mitochondria. Mitochondria play an important role in the metabolism, energy production, signal transduction and life and death cycle of cancer cells. At the same time, the mutation and abnormal expression of mitochondrial genome is also an important factor in cancer development. Therefore, in-depth study of the relationship between mitochondria and cancer is of great significance for revealing the mechanism of cancer and developing new treatment methods.

Aberrant energy metabolism represents one of the most fundamental characteristics exhibited by cancer cells, with the Warburg effect being a typical hallmark observed in numerous malignant tumors [8]. Cancer cells autonomously modulate their metabolic pathways to fulfill augmented bioenergetic and biosynthetic demands while mitigating oxidative stress induced by uncontrolled proliferation [81]. However, it has been confirmed that OXPHOS is significantly up-regulated in most cancer cells instead of being inhibited as anticipated [82]. Moreover, the team led by Yufeng Shi has discovered that Gboxin is a mitochondrial OXPHOS inhibitor specific to tumor stem cells, effectively impeding tumorigenesis and progression [83]. Therefore, the OXPHOS pathway represents a promising target for anti-tumor therapy. However, studies have revealed significant variations in the sensitivity of tumor cells to inhibitors of mitochondrial OXPHOS. Only tumors that heavily rely on mitochondrial OXPHOS as an energy source can be effectively targeted by these inhibitors [83]. Consequently, investigating the targeted regulation of genes associated with OXPHOS in tumor cells and inhibiting the up-regulation of OXPHOS emerge as emerging and potentially efficacious strategies for tumor treatment [84]. The present study at the first time found that, compared with LGG, HGG showed more integrated MRC morphology, by using combined machine learning-based transmission electron microscopy analysis of 7141 mitochondria from 54 resected patients’ glioma. Additionally, through the utilization of mitochondrial OCR detection and MMP detection, we have observed a significant enhancement in both morphology and functionality of MRC within U87MG/U251MG/GSCs by means of DNM1L/DRP1 binding to its receptor FIS1. More importantly, targeted inhibition of DNM1L/DRP1 or FIS1 can effectively attenuate the increase of MRC and OXPHOS, thereby impeding the progression of HGG.

The IMM becomes depressed towards the matrix side, forming the MRC that serves as the primary site for OXPHOS [22]. Consequently, the integrity of the IMM plays a crucial role in OXPHOS within mitochondria [21]. Consistent findings were observed in HGG where a higher proportion of mitochondria exhibited dense and intact respiratory cristae. Our hypothesis suggests that HGG with prominent mitochondrial fission possess an increased number of healthy mitochondria characterized by dense MRC and intact structures, thereby enhancing their metabolic capacity and promoting tumor proliferation and migration. When glucose is lacking and galactose is utilized as a carbon source by GBM cells, there is a reprogramming of GBM metabolism, resulting in an increase in DNM1L/DRP1 expression, enhances OXPHOS activity, decreases glycolysis, and promotes tumor migration [18]. Similar findings were observed in hepatocellular carcinoma cells, where increased DNM1L/DRP1 expression and augmented mitochondrial fission led to elevated OXPHOS levels, thereby promoting tumor proliferation and development [61]. Similarly, we found that in glioma cells, the up-regulation of DNM1L/DRP1 and FIS1 both promoted mitochondrial fission, significantly increased mitochondrial OXPHOS, and ultimately enhanced the proliferation and migration of glioma. Additionally, previous reports have demonstrated that enhanced glycolysis in tumor cells and macrophages elevates pyruvate content, thereby promoting tricarboxylic acid cycle and OXPHOS. This metabolic shift provides malignant tumors with high energy production and resistance [85]. Consistent with these findings, our results also revealed a significant enhancement in glycolysis in glioma cells following the up-regulation of DNM1L/DRP1 and FIS1. Therefore, it can be concluded that the proteins related to mitochondrial fission—DNM1L/DRP1 and FIS1—are closely associated with OXPHOS and play a crucial role in tumorigenesis.

The morphology of mitochondria, highly dynamic organelles, undergoes constant changes in parallel with energy metabolism [80]. Not only GBM cell lines U87MG and U251MG but also glioma stem cells BT25 and BITC exhibit a propensity for excessive mitochondrial fission [14, 18, 86]. Additionally, various other tumor types [10, 12, 54, 61] demonstrate a significant increase in mitochondrial fission, consistent with our observation that HGG display more pronounced mitochondrial fission. This aberrant mitochondrial fission in tumor cells is accompanied by elevated expression of DNM1L/DRP1 [87]. Combined with bioinformatics analysis and mIHC staining, we observed a significantly higher expression of mitochondrial fission-related proteins in HGG compared to LGG. The dynamic equilibrium between mitochondrial fission and fusion is crucial for maintaining cellular physiological activity, and dysfunctional mitochondrial dynamics are closely associated with the initiation and progression of various human malignancies, including lung cancer, breast cancer, and colon cancer [88].

The maintenance of a stable MMP is essential for efficient mitochondrial OXPHOS. Numerous studies have consistently demonstrated that in the majority of tumors, dysregulated mitochondrial dynamics result in alterations in MMP, consequently leading to significant perturbations in OXPHOS [10, 54, 61, 64]. Therefore, aberrant changes in MMP play a crucial role in tumor progression. Additionally, Orian Shirihai discovered that impairment of MRC can also result in a significant reduction in MMP [89]. ROS is a byproduct of OXPHOS, and the heightened metabolic activity leads to an elevated ROS level in cancer cells compared to normal cells, rendering cancer cells more vulnerable to oxidative stress. This phenomenon induces alterations in MMP and ultimately influences the initiation and progression of tumors [65, 90]. The targeted down-regulation of the DNM1L/DRP1-FIS1 axis in our study significantly impacted mitochondrial quality control, thereby influencing ROS production and MMP stability, ultimately inhibiting glioma progression.

The impairment of mitochondria results in a reduction in MMP, which triggers the recruitment of PINK1/PARKIN from the cytoplasm to the OMM, initiating autophagy and regulating mitochondrial quality [91]. Studies have proposed that rapamycin can induce the expression of DNM1L/DRP1 and FIS1 while increasing PINK1 and PARKIN expression to promote mitophagy. This leads to an increase in the number of intact mitochondria within tumor cells and subsequently plays a role in suppressing tumor development [91]. More significantly, DNM1L/DRP1-FIS1 facilitates autophagy, a peripheral mitochondrial fission process that eliminates damaged regions of mitochondria, as reported in Nature [56]. Recent studies have demonstrated that remodeling the MRC augments mitochondrial respiration [21, 22]. After up-regulating DNM1L/DRP1 and FIS1, we observed an increase in PINK1 and PARKIN expression, enhanced autophagy, and an augmented proportion of MRC. This significantly potentiated mitochondrial OXPHOS, ultimately facilitating the proliferation and progression of glioma cells. Therefore, as the malignancy level of glioma cells increases, the elevated levels of DNM1L/DRP1 and FIS1 lead to heightened expression of PINK1/PARKIN, promoting mitochondrial fission and reshaping MRC through autophagy. This resulted in a greater number of mitochondria with improved structural integrity and enhanced functionality that ultimately promote tumor development. However, following up-regulation of MFF, the expression levels of DNM1L/DRP1 and FIS1 were observed to decrease, thereby inhibiting tumor proliferation and progression. This finding provided evidence for distinct roles played by FIS1 and MFF in the regulation of mitochondrial quality. We hypothesize that apart from its involvement as a receptor for DNM1L/DRP1 during mitochondrial division, MFF also interacts with FIS1 to contribute towards quality monitoring and prevention of tumor progression. This finding is consistent with previous reports highlighting the potent anti-cancer activity exhibited by MFF across various tumor models [58]. However, the assessment of mitochondrial dynamics and function in our study was limited to GBM cell lines, and there was a dearth of clinical data support. The effects of DNM1L/DRP1, FIS1 and MFF on complex I-V in the process of OXPHOS necessitate further analysis, and it is imperative to conduct additional investigations and validation through cell metabolomics to determine their potential impact on metabolic reprogramming.

## Conclusions

Our work, along with other studies reported in the literature, supports the notion that the inhibition of DNM1L/DRP1-FIS1 axis affects the remodeling of MRC and limits the aerobic respiration in glioma. Specifically, our study has demonstrated that targeted inhibition of the DNM1L/DRP1-FIS1 axis is essential for regulating mitochondrial division and inhibiting glioma progression. Furthermore, we have elucidated the intricate relationship between different modes of mitochondrial division, MRC remodeling, and OXPHOS in glioma, thereby providing valuable clinical implications for treating gliomas of varying grades.

## Supplementary Information

Supplementary Material 1.

Supplementary Material 2.

Supplementary Material 3.

## Data Availability

The Methods encompass all routine analytical techniques. The paper and Extended data provide data that support the findings of this study, while the corresponding author can furnish the original data upon reasonable request. The electron microscopy mitochondria dataset of glioma and the machine learning-based artificial recognition system employed in this study were collaboratively developed by the Teaching and Experiment Center and the Computer Teaching and Research Department of the The Fourth Military Medical University. The electron microscopy images of glioma tissues from all patients are available for inquiry on the following website: https://oss.fmmutec.com/scientific/Xiaodong%20Li/Electron%20microscope%20image%20of%20patient.zip*.*

## Abbreviations

2-DG: 2-Deoxyglucose
ATG5: Autophagy-related protein 5
bFGF: Basic fibroblast growth factor
CCCP: Carbonyl cyanide 3-chlorophenylhydrazone
CNS: Central nervous system
COCO: Common Objects in Context
CSCs: Cancer stem cells
DMEM: Dulbecco's Modified Eagle's Medium
DMSO: Dimethyl sulfoxide
DNM1L/DRP1: Dynamic-1-like protein
ECAR: Extracellular acidification rate
EGF: Epidermal growth factor
FBS: Fetal bovine serum
FCCP: Carbonyl cyanide 4-(trifluoromethoxy) phenylhydrazone
FIS1: Mitochondrial fission protein 1
FN: False negatives
FP: False positives
FUNDC1: FUN14 domain containing 1
GBM: Glioblastoma
GFP: Green fluorescent protein
GSCs: Glioma stem cells
GTEx: Genotype-Tissue Expression
HGG: High-grade glioma
IMM: Inner mitochondrial membrane
LC3: Microtubule-associated protein 1 light chain 3
LGG: Low-grade glioma
Mask R-CNN: Mask Region-based Convolutional Neural Network
MEM: Minimum Essential Medium
MFF: Mitochondrial fission factor
MFN1/2: Mitochondrial fusion protein 1 and 2
mIHC staining: Multiplex immunohistochemical staining
MMP: Mitochondrial membrane potential
MOI: Multiplicity of infection
MRC: Mitochondrial respiratory cristae
MRGs: Mitochondria-related genes
NAC: N-acetyl-L-cysteine
NCG: NOD/ShiLtJGpt-*Prkdc*^em26Cd52^*Il2rg*^em26Cd22^/Gpt
NEAA: Non-essential amino acid
OCR: Oxygen consumption rate
OMM: Outer mitochondrial membrane
OXPHOS: Oxidative phosphorylation
PBS: Phosphate buffer saline
PE: Phosphatidylethanolamine
PFA: Paraformaldehyde
PINK1: PTEN induced putative kinase 1
ROS: Reactive oxygen species
SGD: Stochastic gradient descent
SOD1: Superoxide Dismutase
SOX2: SRY-related HMG-box 2
STR: Short Tandem Repeat
TBHP: Tert-Butyl Hydroperoxide
TCA cycle: Tricarboxylic acid cycle
TCGA: The Cancer Genome Atlas
TP: True positives
WHO: World Health Organization
