# Supplementary material for: Targeting DNM1L/DRP1-FIS1 axis inhibits high-grade glioma progression by impeding mitochondrial respiratory cristae remodeling
Source: J Exp Clin Cancer Res. 2024 Sep 30;43:273. doi: 10.1186/s13046-024-03194-6 (PMC11440692; doi:10.1186/s13046-024-03194-6)
Supplement: Supplementary file 2 — Supplementary Material 2. [file 13046_2024_3194_MOESM2_ESM.docx]

# Supplementary information:

## Supplementary Figure 1.

We carried out the study aiming at MRC and its OXPHOS outcome in HGG from three aspects. First in green, machine learning-based transmission electron microscopy analysis of 7141 mitochondria from 54 resected patients’ glioma, combined with bioinformatics analysis and mIHC staining of clinical glioma microarray, was used to compare the structural features of MRC between LGG and HGG, then to screen the key genes for this phenomenon. Second in blue, where we modulated the expression of DNM1L/DRP1, FIS1, and MFF in U87MG/U251MG/GSCs, we also administered the DNM1L/DRP1 inhibitor Mdivi-1 to observe the expression of mitochondrial-related molecules. Furthermore, we assessed the OXPHOS capacity of mitochondria by measuring basal respiration and ATP production. We evaluated the mitochondrial state by measuring MMP, ROS, and MRC abundance. Finally, we determined the level of mitophagy by examining the expression levels of autophagy-related molecules and conducting Mito-Keima staining. Third in pink, we studied the effects of DNM1L/DRP1, FIS1, and MFF on glycolysis by ECAR assay, and HGG progression by CCK-8, Transwell, Wound Healing, tumor ball, xenograft and Ki-67 assays in mice.

## Supplementary Figure 2.

**a.** Western blot, expressions of mitochondria-related proteins in the NHAs, U87MG and U251MG. NHAs: Normal human astrocytes.

**b.** Statistical analysis of Western blot.

The data were presented as means ± SD. *P* were calculated by unpaired two-tailed t-test.

## Supplementary Figure 3.

**a.** The CCK-8 assay, detected the proliferation ability of U251MG after regulation of DNM1L (n = 6).

**b.** The Transwell assay, detected the migration ability of U251MG after regulation of DNM1L (n = 6). Scale bars: 200 µm.

**c.** The Wound Healing assay, detected the migration ability of U251MG after regulation of DNM1L (n = 6). Scale bars: 500 µm.

**d.** The CCK-8 assay, detected the proliferation ability of U87MG and U251MG after Mdivi-1 intervention (n = 6).

**e.** The Transwell assay, detected the migration ability of U87MG and U251MG after Mdivi-1 intervention (n = 6). Scale bars: 200 µm.

**f.** The Wound Healing assay, detected the migration ability of U87MG and U251MG after Mdivi-1 intervention (n = 6). Scale bars: 500 µm.

**g.** Western blot, expression level of mitochondria-related proteins in the U251MG when DNM1L was downregulated and overexpressed.

**h.** Statistical analysis of Western blot after regulation of DNM1L.

**i.** Western blot, expressions of mitochondria-related proteins in the U251MG after Mdivi-1 intervention.

**j.** Statistical analysis of Western blot after Mdivi-1 intervention in U251MG.

The data were presented as means ± SD. *P* were calculated by two-way ANOVA (**a, c, d, f**) or unpaired two-tailed t-test (**b, e, h, j**).

## Supplementary Figure 4.

**a.** The CCK-8 assay, detected the proliferation ability of U87MG after up-regulation of FIS1 and MFF (n = 6).

**b.** The Transwell assay, detected the migration ability of U87MG after up-regulation of FIS1 and MFF (n = 6). Scale bars: 200 µm.

**c.** The Wound Healing assay, detected the migration ability of U87MG after up-regulation of FIS1 and MFF (n = 6). Scale bars: 500 µm.

**d. and e.** Glioma cell derived xenograft assay after up-regulation of FIS1 and MFF (n = 6). **P* < 0.05; ***P* < 0.01; ****P* < 0.001; *****P* < 0.0001.

**f.** Representative confocal images of U87MG in OE-FIS1: DAPI (blue), Ki-67 (red) and merge. Scale bars: 50 µm. Quantitative analysis of % of Ki-67⁺ cells (n = 6).

**g.** Representative confocal images of U87MG in OE-MFF: DAPI (blue), Ki-67 (red) and merge. Scale bars: 50 µm. Quantitative analysis of % of Ki-67⁺ cells (n = 6).

The data were presented as means ± SD. *P* were calculated by two-way ANOVA (**a, c, d, e**) or unpaired two-tailed t-test (**b, f, g**).

## Supplementary Figure 5.

**a.** The CCK-8 assay, detected the proliferation ability of U251MG after down-regulation of FIS1 (n = 6).

**b.** The Transwell assay, detected the migration ability of U251MG after down-regulation of FIS1 (n = 6). Scale bars: 200 µm.

**c.** The Wound Healing assay, detected the migration ability of U251MG after down-regulation of FIS1 (n = 6). Scale bars: 500 µm.

**d.** The CCK-8 assay, detected the proliferation ability of U251MG after down-regulation of MFF (n = 6).

**e.** The Transwell assay, detected the migration ability of U251MG after down-regulation of MFF (n = 6). Scale bars: 200 µm.

**f.** The Wound Healing assay, detected the migration ability of U251MG after down-regulation of MFF (n = 6). Scale bars: 500 µm.

**g.** The CCK-8 assay, detected the proliferation ability of U251MG after up-regulation of FIS1 (n = 6).

**h.** The Transwell assay, detected the migration ability of U251MG after up-regulation of FIS1 (n = 6). Scale bars: 200 µm.

**i.** The Wound Healing assay, detected the migration ability of U251MG after up-regulation of FIS1 (n = 6). Scale bars: 500 µm.

**j.** The CCK-8 assay, detected the proliferation ability of U251MG after up-regulation of MFF (n = 6).

**k.** The Transwell assay, detected the migration ability of U251MG after up-regulation of MFF (n = 6). Scale bars: 200 µm.

**l.** The Wound Healing assay, detected the migration ability of U251MG after up-regulation of MFF (n = 6). Scale bars: 500 µm.

**m.** Western blot, expressions of mitochondria-related proteins in the U251MG when FIS1 was downregulated and overexpressed.

**n.** Statistical analysis of Western blot after regulation of FIS1.

**o.** Western blot, expressions of mitochondria-related proteins in the U251MG when MFF was downregulated and overexpressed.

**p.** Statistical analysis of Western blot after regulation of MFF.

The data were presented as means ± SD. *P* were calculated by two-way ANOVA (**a, c, d, f, g, i, j, l**) or unpaired two-tailed t-test (**b, e, h, k, o, p**).

## Supplementary Figure 6.

**a. and b.** Immunofluorescence staining unveils the expression of GSC markers. Scale bars: 200 µm.

## Supplementary Figure 7.

**a.** Western blot, expressions of mitochondria-related proteins in the GSCs when DNM1L or FIS1 was up-regulated.

**b.** Statistical analysis of Western blot after up-regulation of DNM1L or FIS1.

**c.** Representative images of electron microscopy after up-regulation of DNM1L or FIS1 (GSCs-OE-NC, n=6; GSCs-OE-DNM1L, n = 6; GSCs-OE-FIS1, n = 6). Scale bars: 0.5

µm.

**d.** Statistical analysis of the mitochondrial size, density and shape after up-regulation of DNM1L or FIS1 (GSCs-OE-NC, n=180; GSCs-OE-DNM1L, n=301; GSCs-OE-FIS1, n = 207).

**e.** The CCK-8 assay, detected the proliferation ability of GSCs after OE-DNM1L and OE-FIS1 (n = 6).

f. Representative images and statistical analysis of tumor spheres after up-regulation of DNM1L or FIS1 (GSCs-OE-NC, n=6; GSCs-OE-DNM1L, n = 6; GSCs-OE-FIS1, n = 6). Scale bars: 200 µm.

**g.** Glioma cell derived xenograft assay after OE-DNM1L or OE-FIS1 (n = 6). **P* < 0.05; ***P* < 0.01; ****P* < 0.001; *****P* < 0.0001.

**h.** Representative confocal images of GSCs after OE-DNM1L or OE-FIS1: DAPI (blue), Ki-67 (red) and merge. Scale bars: 50 µm. Quantitative analysis of % of Ki-67⁺ cells (n = 6).

The data were presented as means ± SD. *P* were calculated by unpaired two-tailed t-test (**b, h**), Mann-Whitney U test (**d**) or two-way ANOVA (**e, g**).

## Supplementary Figure 8.

**a.** Western blot, expressions of mitochondria-related proteins in the GSCs when MFF was regulated.

**b.** Statistical analysis of Western blot after down-regulation of MFF.

**c.** Representative images of electron microscopy after down-regulation of MFF (GSCs-sh-NC, n=6; GSCs-sh-MFF, n = 6; GSCs-OE-NC, n = 6; GSCs-OE-MFF, n = 6). Scale bars: 0.5 µm.

**d.** Statistical analysis of the mitochondrial size, density and shape after regulation of MFF (GSCs-sh-NC, n=272; GSCs-sh-MFF, n = 269; GSCs-OE-NC, n = 180; GSCs-OE-MFF, n = 223).

**e.** The CCK-8 assay, detected the proliferation ability of GSCs after sh-MFF and OE-MFF (n = 6).

f. Representative images and statistical analysis of tumor spheres after regulation of MFF (GSCs-sh-NC, n=6; GSCs-sh-MFF, n = 6; GSCs-OE-NC, n = 6; GSCs-OE-MFF, n = 6). Scale bars: 200 µm.

**g.** Glioma cell derived xenograft assay after sh-MFF and OE-MFF (n = 6). **P* < 0.05; ***P* < 0.01; ****P* < 0.001; *****P* < 0.0001.

**h.** Representative confocal images of GSCs after sh-MFF and OE-MFF: DAPI (blue), Ki-67 (red) and merge. Scale bars: 50 µm. Quantitative analysis of % of Ki-67⁺ cells (n = 6).

The data were presented as means ± SD. *P* were calculated by unpaired two-tailed t-test (**b, h**), Mann-Whitney U test (**d**) or two-way ANOVA (**e, g**).

## Supplementary Figure 9.

**a.** Seahorse XF Cell Mitochondrial Stress Test, statistical analysis of various indices representing the mitochondria OXPHOS ability of NHAs, U87MG and U251MG (n = 5).

**b.** Seahorse XF Glycolysis Rate Assay, statistical analysis of various indices representing the mitochondria glycolysis ability of NHAs, U87MG and U251MG (n = 5).

**c.** Seahorse XF Cell Mitochondrial Stress Test, statistical analysis of various indices representing the mitochondria OXPHOS ability after regulation of DNM1L (n = 5).

**d.** Seahorse XF Glycolysis Rate Assay, statistical analysis of various indices representing the glycolysis ability after regulation of DNM1L (n = 5).

**e.** Seahorse XF Cell Mitochondrial Stress Test, statistical analysis of various indices representing the mitochondria OXPHOS ability after regulation of FIS1 (n = 5).

**f.** Seahorse XF Glycolysis Rate Assay, statistical analysis of various indices representing the glycolysis ability after regulation of FIS1 (n = 5).

**g.** Seahorse XF Cell Mitochondrial Stress Test, statistical analysis of various indices representing the mitochondria OXPHOS ability after regulation of MFF (n = 5).

**h.** Seahorse XF Glycolysis Rate Assay, statistical analysis of various indices representing the glycolysis ability after regulation of MFF (n = 5).

The data were presented as means ± SD. *P* were calculated by unpaired two-tailed t-test.

## Supplementary Figure 10.

**a.** Seahorse XF Cell Mitochondrial Stress Test, statistical analysis of various indices representing the mitochondria OXPHOS ability after regulation of DNM1L (n = 5).

**b.** Seahorse XF Glycolysis Rate Assay, statistical analysis of various indices representing the glycolysis ability after regulation of DNM1L (n = 5).

**c.** Seahorse XF Cell Mitochondrial Stress Test, statistical analysis of various indices representing the mitochondria OXPHOS ability after regulation of FIS1 (n = 5).

**d.** Seahorse XF Glycolysis Rate Assay, statistical analysis of various indices representing the glycolysis ability after regulation of FIS1 (n = 5).

**e.** Seahorse XF Cell Mitochondrial Stress Test, statistical analysis of various indices representing the mitochondria OXPHOS ability after regulation of MFF (n = 5).

**f.** Seahorse XF Glycolysis Rate Assay, statistical analysis of various indices representing the glycolysis ability after regulation of MFF (n = 5).

The data were presented as means ± SD. *P* were calculated by unpaired two-tailed t-test.

## Supplementary Figure 11.

**a.** Statistical analysis of OCR/ECAR after down-regulation of DNM1L in U87MG, U251MG and GSCs (n = 5).

**b.** Statistical analysis of OCR/ECAR after up-regulation of DNM1L in U87MG, U251MG and GSCs (n = 5).

**c.** Statistical analysis of OCR/ECAR after down-regulation of FIS1 in U87MG, U251MG and GSCs (n = 5).

**d.** Statistical analysis of OCR/ECAR after up-regulation of FIS1 in U87MG, U251MG and GSCs (n = 5).

**e.** Statistical analysis of OCR/ECAR after down-regulation of MFF in U87MG, U251MG and GSCs (n = 5).

**f.** Statistical analysis of OCR/ECAR after up-regulation of MFF in U87MG, U251MG and GSCs (n = 5).

The data were presented as means ± SD. *P* were calculated by unpaired two-tailed t-test.

## Supplementary Figure 12.

**a.** Detected the MMP of NHAs, U87MG and U251MG using JC-1 (n = 9).

**b.** Detected the ROS of NHAs, U87MG and U251MG using DCFH-DA (n = 9).

**c.** Detected the MMP of U251MG after down-regulation of DNM1L by TMRE kit, the mean fluorescence intensity of positive cells was measured. (n = 6).

**d.** Detected the MMP of U251MG after overexpression of DNM1L by TMRE kit, the mean fluorescence intensity of positive cells was measured (n = 6).

**e.** Detected the ROS of U251MG after down-regulation of DNM1L by CellROX®Deep Red kit, the mean fluorescence intensity of positive cells was measured (n = 6).

**f.** Detected the ROS of U251MG after overexpression of DNM1L CellROX®Deep Red kit, the mean fluorescence intensity of positive cells was measured (n = 6).

**g.** Detected the MMP of U251MG after down-regulation of FIS1 by TMRE kit, the mean fluorescence intensity of positive cells was measured (n = 6).

**h.** Detected the MMP of U251MG after overexpression of FIS1 by TMRE kit, the mean fluorescence intensity of positive cells was measured (n = 6).

**i.** Detected the ROS of U251MG after down-regulation of FIS1 by CellROX®Deep Red kit, the mean fluorescence intensity of positive cells was measured (n = 6).

**j.** Detected the ROS of U251MG after overexpression of FIS1 CellROX®Deep Red kit, the mean fluorescence intensity of positive cells was measured (n = 6).

**k.** Detected the MMP of U251MG after down-regulation of MFF by TMRE kit, the mean fluorescence intensity of positive cells was measured. (n = 6).

**l.** Detected the MMP of U251MG after overexpression of MFF by TMRE kit, the mean fluorescence intensity of positive cells was measured (n = 6).

**m.** Detected the ROS of U251MG after down-regulation of MFF by CellROX®Deep Red kit, the mean fluorescence intensity of positive cells was measured (n = 6).

**n.** Detected the ROS of U87MG after overexpression of MFF CellROX®Deep Red kit, the mean fluorescence intensity of positive cells was measured (n = 6).

**o.** Detected the MMP of U87MG after Mdivi-1 intervention using JC-1 (n = 6).

**p.** Detected the ROS of U87MG after Mdivi-1 intervention using DCFH-DA (n = 6).

**q.** The electron microscopy image depicted mitochondria. Scale bars: 0.5 µm.

**r.** Statistical analysis of cristae abundance/mitochondrial area.

**s.** Detected the MMP of U251MG after Mdivi-1 intervention using JC-1 (n = 6).

**t.** Detected the ROS of U251MG after Mdivi-1 intervention using DCFH-DA (n = 6).

The data were presented as means ± SD. *P* were calculated by ordinary one-way ANOVA (**a, b**), unpaired two-tailed t-test (**c-p, s, t**) or Mann-Whitney U test (**q, r**).

## Supplementary Figure 13.

**a. and b.** Detected the MMP and ROS of GSCs after down-regulation of DNM1L, the mean fluorescence intensity of positive cells was measured. (n = 6).

**c.** The cross-section of the MRC was highlighted in green in the electron microscope images, and a statistical analysis was conducted to determine the extent of MRC occupancy. Scale bars: 0.5 µm.

**d. and e.** Detected the MMP and ROS of GSCs after overexpression of DNM1L, the mean fluorescence intensity of positive cells was measured (n = 6).

**f.** The details depicted in the picture align with those presented in (**c**).

**g. and h.** Detected the MMP and ROS of GSCs after down-regulation of FIS1, the mean fluorescence intensity of positive cells was measured (n = 6).

**i.** The details depicted in the picture align with those presented in (**c**).

**j. and k.** Detected the MMP and ROS of GSCs after overexpression of FIS1, the mean fluorescence intensity of positive cells was measured (n = 6).

**l.** The details depicted in the picture align with those presented in (**c**).

**m. and n.** Detected the MMP and ROS of GSCs after down-regulation of MFF, the mean fluorescence intensity of positive cells was measured. (n = 6).

**o.** The details depicted in the picture align with those presented in (**c**).

**p. and q.** Detected the MMP and ROS of GSCs after overexpression of MFF, the mean fluorescence intensity of positive cells was measured (n = 6).

**r.** The details depicted in the picture align with those presented in (**c**).

The data were presented as means ± SD. *P* were calculated by unpaired two-tailed t-test (**a, b, d, e, g, h, j, k, m, n, p, q**) or Mann-Whitney U test (**c, f, i, l, o, r**).

## Supplementary Figure 14.

**a.** Western blot, expressions of mitochondria-related proteins in the U251MG when DNM1L was downregulated and overexpressed.

**b.** Statistical analysis of Western blot after regulation of DNM1L.

**c.** Representative confocal images of U251MG in sh-DNM1L and OE-DNM1L: Mito-Keima (440 nm, green), Mito-Keima (561 nm, red) and merge. Scale bars: 50 µm.

**d.** Statistical analysis of red/green (OE-DNM1L: n = 152; sh-DNM1L: n = 114; OE-NC: n = 120; sh-NC: n = 119).

**e.** Western blot, expressions of mitochondria-related proteins in the U251MG when FIS1 was downregulated and overexpressed.

**f.** Statistical analysis of Western blot after regulation of FIS1.

**g.** Representative confocal images of U251MG in sh-FIS1 and OE-FIS1: Mito-Keima (440 nm, green), Mito-Keima (561 nm, red) and merge. Scale bars: 50 µm.

**h.** Statistical analysis of red/green (OE-FIS1: n = 112; sh-FIS1: n = 133; OE-NC: n = 120; sh-NC: n = 119).

**i.** Western blot, expressions of mitochondria-related proteins in the U251MG when MFF was downregulated and overexpressed.

**j.** Statistical analysis of Western blot after regulation of MFF.

**k.** Representative confocal images of U251MG in sh-MFF and OE-MFF: Mito-Keima (440 nm, green), Mito-Keima (561 nm, red) and merge. Scale bars: 50 µm.

**l.** Statistical analysis of red/green (OE-MFF: n = 112; sh-MFF: n = 119; OE-NC: n = 120; sh-NC: n = 119).

The data were presented as means ± SD. *P* were calculated by unpaired two-tailed t-test (**b, f, j**) or Mann-Whitney U test (**d, h, l**).

## Supplementary Figure 15.

**a.** Western blot, expressions of mitochondria-related proteins in the GSCs when DNM1L was downregulated and overexpressed.

**b.** Statistical analysis of Western blot after regulation of DNM1L.

**c.** Representative confocal images of GSCs in sh-DNM1L and OE-DNM1L: Mito-Keima (440 nm, green), Mito-Keima (561 nm, red) and merge. Scale bars: 50 µm.

**d.** Quantitative analysis of red/green (OE-DNM1L: n = 115; sh-DNM1L: n = 126; OE-NC: n = 108; sh-NC: n = 107).

**e.** Western blot, expressions of mitochondria-related proteins in the GSCs when FIS1 was downregulated and overexpressed.

**f.** Statistical analysis of Western blot after regulation of FIS1.

**g.** Representative confocal images of GSCs in sh-FIS1 and OE-FIS1: Mito-Keima (440 nm, green), Mito-Keima (561 nm, red) and merge. Scale bars: 50 µm.

**h.** Quantitative analysis of red/green (OE-FIS1: n = 120; sh-FIS1: n = 126; OE-NC: n = 108; sh-NC: n = 107).

**i.** Western blot, expressions of mitochondria-related proteins in the GSCs when MFF was downregulated and overexpressed.

**j.** Statistical analysis of Western blot after regulation of MFF.

**k.** Representative confocal images of GSCs in sh-MFF and OE-MFF: Mito-Keima (440 nm, green), Mito-Keima (561 nm, red) and merge. Scale bars: 50 µm.

**l.** Quantitative analysis of red/green (OE-MFF: n = 95; sh-MFF: n = 132; OE-NC: n = 108; sh-NC: n = 107).

The data were presented as means ± SD. *P* were calculated by unpaired two-tailed t-test (**b, f, j**) or Mann-Whitney U test (**d, h, l**).

## Supplementary Table 2. Multiplex Immunohistochemistry

| Cat No. | Lot No. | Point Location | | Pathological Type | Grade | |
| --- | --- | --- | --- | --- | --- | --- |
| HBraG125PG01 | XT18-011 | A01 | | cerebral cortex | normal | |
| HBraG125PG01 | XT18-011 | A02 | | cerebral cortex | normal | |
| HBraG125PG01 | XT18-011 | A03 | | cerebral cortex | normal | |
| HBraG125PG01 | XT18-011 | A04 | | ganglioglioma | 1 | |
| HBraG125PG01 | XT18-011 | A05 | | pilocytic astrocytoma | 1 | |
| HBraG125PG01 | XT18-011 | A06 | | pilocytic astrocytoma | 1 | |
| HBraG125PG01 | XT18-011 | A07 | | pilocytic astrocytoma | 1 | |
| HBraG125PG01 | XT18-011 | A08 | | pilocytic astrocytoma | 1 | |
| HBraG125PG01 | XT18-011 | A09 | | oligodendroglioma | 2 | |
| HBraG125PG01 | XT18-011 | A10 | | oligodendroglioma | 2 | |
| HBraG125PG01 | XT18-011 | A11 | | oligodendroglioma | 2 | |
| HBraG125PG01 | XT18-011 | A12 | | pleomorphic xanthoastrocytoma | 2 | |
| HBraG125PG01 | XT18-011 | A13 | | pleomorphic xanthoastrocytoma | 2 | |
| HBraG125PG01 | XT18-011 | A14 | | diffuse astrocytoma | 2 | |
| HBraG125PG01 | XT18-011 | B01 | | diffuse astrocytoma | 2 | |
| HBraG125PG01 | XT18-011 | B02 | | diffuse astrocytoma | 2 | |
| HBraG125PG01 | XT18-011 | B03 | | diffuse astrocytoma | 2 | |
| HBraG125PG01 | XT18-011 | B04 | | diffuse astrocytoma | 2 | |
| HBraG125PG01 | XT18-011 | B05 | | diffuse astrocytoma | 2 | |
| HBraG125PG01 | XT18-011 | B06 | | diffuse astrocytoma | 2 | |
| HBraG125PG01 | XT18-011 | B07 | | diffuse astrocytoma | 2 | |
| HBraG125PG01 | XT18-011 | B08 | | diffuse astrocytoma | 2 | |
| HBraG125PG01 | XT18-011 | B09 | | diffuse astrocytoma | 2 | |
| HBraG125PG01 | XT18-011 | B10 | | diffuse astrocytoma | 2 | |
| HBraG125PG01 | XT18-011 | B11 | | diffuse astrocytoma | 2 | |
| HBraG125PG01 | XT18-011 | B12 | | diffuse astrocytoma | 2 | |
| HBraG125PG01 | XT18-011 | B13 | | diffuse astrocytoma | 2 | |
| HBraG125PG01 | XT18-011 | B14 | | diffuse astrocytoma | 2 | |
| HBraG125PG01 | XT18-011 | C01 | | diffuse astrocytoma | 2 | |
| HBraG125PG01 | XT18-011 | C02 | | diffuse astrocytoma | 2 | |
| HBraG125PG01 | XT18-011 | C03 | | oligoastrocytoma | 2 | |
| HBraG125PG01 | XT18-011 | C04 | | oligoastrocytoma | 2 | |
| HBraG125PG01 | XT18-011 | C05 | | oligoastrocytoma | 2 | |
| HBraG125PG01 | XT18-011 | C06 | | oligoastrocytoma | 2 | |
| HBraG125PG01 | XT18-011 | C07 | | oligoastrocytoma | 2 | |
| HBraG125PG01 | XT18-011 | C08 | | oligoastrocytoma | 2 | |
| HBraG125PG01 | XT18-011 | C09 | | oligoastrocytoma | 2 | |
| HBraG125PG01 | XT18-011 | C10 | | oligoastrocytoma | 2 | |
| HBraG125PG01 | XT18-011 | C11 | | oligoastrocytoma | 2 | |
| HBraG125PG01 | XT18-011 | C12 | | oligoastrocytoma | 2 | |
| HBraG125PG01 | XT18-011 | C13 | | oligoastrocytoma | 2 | |
| HBraG125PG01 | XT18-011 | C14 | | oligoastrocytoma | 2 | |
| HBraG125PG01 | XT18-011 | D01 | | oligoastrocytoma, some anaplastic | 3 | |
| HBraG125PG01 | XT18-011 | D02 | | anaplastic oligodendroglioma | 3 | |
| HBraG125PG01 | XT18-011 | D03 | | anaplastic astrocytoma | 3 | |
| HBraG125PG01 | XT18-011 | D04 | | anaplastic astrocytoma | 3 | |
| HBraG125PG01 | XT18-011 | D05 | | anaplastic astrocytoma | 3 | |
| HBraG125PG01 | XT18-011 | D06 | | anaplastic astrocytoma | 3 | |
| HBraG125PG01 | XT18-011 | D07 | | anaplastic astrocytoma | 3 | |
| HBraG125PG01 | XT18-011 | D08 | | anaplastic astrocytoma | 3 | |
| HBraG125PG01 | XT18-011 | D09 | | anaplastic oligodendroglioma | 3 | |
| HBraG125PG01 | XT18-011 | D10 | | anaplastic oligodendroglioma | 3 | |
| HBraG125PG01 | XT18-011 | D11 | | anaplastic oligodendroglioma | 3 | |
| HBraG125PG01 | XT18-011 | D12 | | anaplastic oligodendroglioma | 3 | |
| HBraG125PG01 | XT18-011 | D13 | | anaplastic astrocytoma | 3 | |
| HBraG125PG01 | XT18-011 | D14 | | anaplastic astrocytoma | 3 | |
| HBraG125PG01 | XT18-011 | E01 | | anaplastic astrocytoma | 3 | |
| HBraG125PG01 | XT18-011 | E02 | | anaplastic astrocytoma | 3 | |
| HBraG125PG01 | XT18-011 | E03 | | anaplastic astrocytoma | 3 | |
| HBraG125PG01 | XT18-011 | E04 | | anaplastic astrocytoma | 3 | |
| HBraG125PG01 | XT18-011 | E05 | | anaplastic oligoastrocytoma | 3 | |
| HBraG125PG01 | XT18-011 | E06 | | anaplastic astrocytoma | 3 | |
| HBraG125PG01 | XT18-011 | E07 | | anaplastic astrocytoma | 3 | |
| HBraG125PG01 | XT18-011 | E08 | | anaplastic astrocytoma | 3 | |
| HBraG125PG01 | XT18-011 | E09 | | anaplastic astrocytoma | 3 | |
| HBraG125PG01 | XT18-011 | E10 | | anaplastic astrocytoma | 3 | |
| HBraG125PG01 | XT18-011 | E11 | | anaplastic astrocytoma | 3 | |
| HBraG125PG01 | XT18-011 | E12 | | anaplastic astrocytoma | 3 | |
| HBraG125PG01 | XT18-011 | E13 | | anaplastic astrocytoma | 3 | |
| HBraG125PG01 | XT18-011 | E14 | | anaplastic oligodendroglioma | 3 | |
| HBraG125PG01 | XT18-011 | F01 | | anaplastic astrocytoma, glioblastoma | 4 | |
| HBraG125PG01 | XT18-011 | F02 | | glioblastoma | 4 | |
| HBraG125PG01 | XT18-011 | F03 | | glioblastoma | 4 | |
| HBraG125PG01 | XT18-011 | F04 | | glioblastoma | 4 | |
| HBraG125PG01 | XT18-011 | F05 | | glioblastoma | 4 | |
| HBraG125PG01 | XT18-011 | F06 | | glioblastoma | 4 | |
| HBraG125PG01 | XT18-011 | F07 | | glioblastoma | 4 | |
| HBraG125PG01 | XT18-011 | F08 | | glioblastoma | 4 | |
| HBraG125PG01 | XT18-011 | F09 | | glioblastoma | 4 | |
| HBraG125PG01 | XT18-011 | F10 | | glioblastoma | 4 | |
| HBraG125PG01 | XT18-011 | F11 | | glioblastoma | 4 | |
| HBraG125PG01 | XT18-011 | F12 | | glioblastoma | 4 | |
| HBraG125PG01 | XT18-011 | F13 | | glioblastoma | 4 | |
| HBraG125PG01 | XT18-011 | F14 | | glioblastoma | 4 | |
| HBraG125PG01 | XT18-011 | G01 | | glioblastoma | 4 | |
| HBraG125PG01 | XT18-011 | G02 | | glioblastoma | 4 | |
| HBraG125PG01 | XT18-011 | G03 | | glioblastoma | 4 | |
| HBraG125PG01 | XT18-011 | G04 | | glioblastoma | 4 | |
| HBraG125PG01 | XT18-011 | G05 | | glioblastoma | 4 | |
| HBraG125PG01 | XT18-011 | G06 | | glioblastoma | 4 | |
| HBraG125PG01 | XT18-011 | G07 | | glioblastoma | 4 | |
| HBraG125PG01 | XT18-011 | G08 | | glioblastoma | 4 | |
| HBraG125PG01 | XT18-011 | G09 | | glioblastoma | 4 | |
| HBraG125PG01 | XT18-011 | G10 | | glioblastoma | 4 | |
| HBraG125PG01 | XT18-011 | G11 | | glioblastoma | 4 | |
| HBraG125PG01 | XT18-011 | G12 | | glioblastoma | 4 | |
| HBraG125PG01 | XT18-011 | G13 | | glioblastoma | 4 | |
| HBraG125PG01 | XT18-011 | G14 | | glioblastoma | 4 | |
| HBraG125PG01 | XT18-011 | H01 | | glioblastoma | 4 | |
| HBraG125PG01 | XT18-011 | H02 | | glioblastoma | 4 | |
| HBraG125PG01 | XT18-011 | H03 | | glioblastoma | 4 | |
| HBraG125PG01 | XT18-011 | H04 | | glioblastoma | 4 | |
| HBraG125PG01 | XT18-011 | H05 | | glioblastoma | 4 | |
| HBraG125PG01 | XT18-011 | H06 | | glioblastoma | 4 | |
| HBraG125PG01 | XT18-011 | H07 | | glioblastoma | 4 | |
| HBraG125PG01 | XT18-011 | H08 | | glioblastoma | 4 | |
| HBraG125PG01 | XT18-011 | H09 | | glioblastoma | 4 | |
| HBraG125PG01 | XT18-011 | H10 | | glioblastoma | 4 | |
| HBraG125PG01 | XT18-011 | H11 | | glioblastoma | 4 | |
| HBraG125PG01 | XT18-011 | H12 | | glioblastoma | 4 | |
| HBraG125PG01 | XT18-011 | H13 | | glioblastoma | 4 | |
| HBraG125PG01 | XT18-011 | H14 | | glioblastoma | 4 | |
| HBraG125PG01 | XT18-011 | I01 | | glioblastoma | 4 | |
| HBraG125PG01 | XT18-011 | I02 | | glioblastoma | 4 | |
| HBraG125PG01 | XT18-011 | I03 | | glioblastoma | 4 | |
| HBraG125PG01 | XT18-011 | I04 | | glioblastoma | 4 | |
| HBraG125PG01 | XT18-011 | I05 | | glioblastoma | 4 | |
| HBraG125PG01 | XT18-011 | I06 | | glioblastoma | 4 | |
| HBraG125PG01 | XT18-011 | I07 | | glioblastoma | 4 | |
| HBraG125PG01 | XT18-011 | I08 | | glioblastoma | 4 | |
| HBraG125PG01 | XT18-011 | I09 | | glioblastoma | 4 | |
| HBraG125PG01 | XT18-011 | I10 | | glioblastoma | 4 | |
| HBraG125PG01 | XT18-011 | I11 | | glioblastoma | 4 | |
| HBraG125PG01 | XT18-011 | I12 | | glioblastoma | 4 | |
| HBraG125PG01 | XT18-011 | I13 | | glioblastoma, gliosarcoma | 4 | |
| HBra-Gli060PG-01 | XT15-005 | A1 | | cerebral cortex | normal | |
| HBra-Gli060PG-01 | XT15-005 | A2 | | cerebral cortex | normal | |
| HBra-Gli060PG-01 | XT15-005 | A3 | | cerebral cortex | normal | |
| HBra-Gli060PG-01 | XT15-005 | A4 | | astrocytoma | 1 | |
| HBra-Gli060PG-01 | XT15-005 | A5 | | pilocytic astrocytoma | 1 | |
| HBra-Gli060PG-01 | XT15-005 | A6 | | pilocytic astrocytoma | 1 | |
| HBra-Gli060PG-01 | XT15-005 | A7 | | astrocytoma | 2 | |
| HBra-Gli060PG-01 | XT15-005 | A8 | | astrocytoma | 2 | |
| HBra-Gli060PG-01 | XT15-005 | A9 | | astrocytoma | 2 | |
| HBra-Gli060PG-01 | XT15-005 | A10 | | astrocytoma | 2 | |
| HBra-Gli060PG-01 | XT15-005 | B1 | | astrocytoma | 2 | |
| HBra-Gli060PG-01 | XT15-005 | B2 | | diffuse astrocytoma | 2 | |
| HBra-Gli060PG-01 | XT15-005 | B3 | | diffuse astrocytoma | 2 | |
| HBra-Gli060PG-01 | XT15-005 | B4 | | diffuse astrocytoma | 2 | |
| HBra-Gli060PG-01 | XT15-005 | B5 | | diffuse astrocytoma | 2 | |
| HBra-Gli060PG-01 | XT15-005 | B6 | | astrocytoma | 3 | |
| HBra-Gli060PG-01 | XT15-005 | B7 | | anaplastic astrocytoma | 3 | |
| HBra-Gli060PG-01 | XT15-005 | B8 | | anaplastic astrocytoma | 3 | |
| HBra-Gli060PG-01 | XT15-005 | B9 | | anaplastic astrocytoma | 3 | |
| HBra-Gli060PG-01 | XT15-005 | B10 | | anaplastic astrocytoma | 3 | |
| HBra-Gli060PG-01 | XT15-005 | C1 | | anaplastic astrocytoma | 3 | |
| HBra-Gli060PG-01 | XT15-005 | C2 | | anaplastic astrocytoma | 3 | |
| HBra-Gli060PG-01 | XT15-005 | C3 | | anaplastic astrocytoma | 3 | |
| HBra-Gli060PG-01 | XT15-005 | C4 | | anaplastic astrocytoma | 3 | |
| HBra-Gli060PG-01 | XT15-005 | C5 | | anaplastic astrocytoma, localized glioblastoma | 3-4 | |
| HBra-Gli060PG-01 | XT15-005 | C6 | | anaplastic astrocytoma, localized glioblastoma | 3-4 | |
| HBra-Gli060PG-01 | XT15-005 | C7 | | glioblastoma, localized astrocytoma | 4 | |
| HBra-Gli060PG-01 | XT15-005 | C8 | | glioblastoma multiforme | 4 | |
| HBra-Gli060PG-01 | XT15-005 | C9 | | glioblastoma multiforme | 4 | |
| HBra-Gli060PG-01 | XT15-005 | C10 | | glioblastoma multiforme | 4 | |
| HBra-Gli060PG-01 | XT15-005 | D1 | | glioblastoma multiforme | 4 | |
| HBra-Gli060PG-01 | XT15-005 | D2 | | glioblastoma multiforme, glioblastoma | 4 | |
| HBra-Gli060PG-01 | XT15-005 | D3 | | glioblastoma | 4 | |
| HBra-Gli060PG-01 | XT15-005 | D4 | | glioblastoma | 4 | |
| HBra-Gli060PG-01 | XT15-005 | D5 | | glioblastoma | 4 | |
| HBra-Gli060PG-01 | XT15-005 | D6 | | glioblastoma | 4 | |
| HBra-Gli060PG-01 | XT15-005 | D7 | | glioblastoma | 4 | |
| HBra-Gli060PG-01 | XT15-005 | D8 | | glioblastoma | 4 | |
| HBra-Gli060PG-01 | XT15-005 | D9 | | glioblastoma | 4 | |
| HBra-Gli060PG-01 | XT15-005 | D10 | | glioblastoma | 4 | |
| HBra-Gli060PG-01 | XT15-005 | E1 | | glioblastoma | 4 | |
| HBra-Gli060PG-01 | XT15-005 | E2 | | glioblastoma | 4 | |
| HBra-Gli060PG-01 | XT15-005 | E3 | glioblastoma | | | 4 |
| HBra-Gli060PG-01 | XT15-005 | E4 | glioblastoma | | | 4 |
| HBra-Gli060PG-01 | XT15-005 | E5 | glioblastoma | | | 4 |
| HBra-Gli060PG-01 | XT15-005 | E6 | glioblastoma | | | 4 |
| HBra-Gli060PG-01 | XT15-005 | E7 | glioblastoma | | | 4 |
| HBra-Gli060PG-01 | XT15-005 | E8 | glioblastoma | | | 4 |
| HBra-Gli060PG-01 | XT15-005 | E9 | glioblastoma | | | 4 |
| HBra-Gli060PG-01 | XT15-005 | E10 | glioblastoma | | | 4 |
| HBra-Gli060PG-01 | XT15-005 | F1 | glioblastoma | | | 4 |
| HBra-Gli060PG-01 | XT15-005 | F2 | glioblastoma | | | 4 |
| HBra-Gli060PG-01 | XT15-005 | F3 | glioblastoma | | | 4 |
| HBra-Gli060PG-01 | XT15-005 | F4 | glioblastoma | | | 4 |
| HBra-Gli060PG-01 | XT15-005 | F5 | glioblastoma | | | 4 |
| HBra-Gli060PG-01 | XT15-005 | F6 | glioblastoma | | | 4 |
| HBra-Gli060PG-01 | XT15-005 | F7 | glioblastoma | | | 4 |
| HBra-Gli060PG-01 | XT15-005 | F8 | glioblastoma | | | 4 |
| HBra-Gli060PG-01 | XT15-005 | F9 | glioblastoma | | | 4 |
| HBra-Gli060PG-01 | XT15-005 | F10 | glioblastoma | | | 4 |
