# Supplementary material for: Targeting DNM1L/DRP1-FIS1 axis inhibits high-grade glioma progression by impeding mitochondrial respiratory cristae remodeling
Source: J Exp Clin Cancer Res. 2024 Sep 30;43:273. doi: 10.1186/s13046-024-03194-6 (PMC11440692; doi:10.1186/s13046-024-03194-6)
Supplement: Supplementary file 1 — Supplementary Material 1. [file 13046_2024_3194_MOESM1_ESM.docx]

Title: Targeting DNM1L/DRP1-FIS1 axis inhibits high-grade glioma progression by impeding mitochondrial respiratory cristae remodeling

**Response to Editor**

Dear Editor,

On behalf of all contributing authors, I would like to express our sincere gratitude to you for giving us the opportunity to revise our manuscript. We also extend our heartfelt thanks to the editor and reviewers for their positive and constructive comments and suggestions on our manuscript titled "Targeting the DRP1/FIS1 axis through inhibiting mitochondrial crista remodeling to suppress the progression of high-grade glioma (ID: JECC-D-24-01360)". The suggestions provided are of great value in improving our work quality. We not only carefully reviewed each suggestion but also had in-depth discussions and implemented them. In response to the reviewer's comments, we made extensive revisions to the manuscript. We have clearly marked the revised content with yellow highlights in the manuscript for your review and comparison.

We sincerely thank you and the reviewers for your comments on our manuscript. Please review the revised manuscript in the attached file, and we look forward to your valuable feedback. Thank you and best regards.

Yours sincerely,

Corresponding author Name: Yayun Wang

E-mail: wangyy@fmmu.edu.cn

**Response to Reviewer #3**

**REVIEWER COMMENTS**

**Reviewer #3 (Remarks to the Author):**

In the revised manuscript, the authors have answered many of my initial queries and the main scientific message of the study is clearer in this version. They have done a great job of including additional data requested by the reviewers, such as new in vivo and in vitro results. In addition, the authors addressed most of the concerns regarding figures and description of the results, improving the accuracy and overall quality of the manuscript. However, I would suggest to the authors some further concerns and comments, which should be addressed for the manuscript to be suitable for publication.

## Specific comments:

### 1. For a more comprehensive description of the data showed in Figure 10 and Supplementary Figures 14-15, the authors should include the description of the results of the Western blot investigating the levels of the autophagy markers (ATG5, LCR-II, P62), as they reported in the answer to my specific comment on this point.

**[Response]**

We sincerely appreciate your time and insightful comments regarding our manuscript. In response to your observation concerning the insufficient detail in the description of autophagy-related molecular changes reflected in the Western blot results, we have conducted a comprehensive analysis and included the pertinent information. Below are the primary modifications we have implemented.

**Please check the lines 983 - 1029.**

“In the course of investigating the autophagy mechanism in glioma cells, Western blot analysis unveiled a pivotal finding: sh-DNM1L treatment led to a significant down-regulation of ATG5 and LC3-II protein expression in glioma cells, concurrently accompanied by a pronounced increase in p62 protein levels. This expression profile strongly indicated that autophagic flux was markedly inhibited, as p62 accumulation was frequently regarded as an indicator of compromised autophagic degradation. Further examination of the mitophagy pathway revealed that both ubiquitin-dependent markers PINK1 and PARKIN, along with non-ubiquitin-dependent markers NIX and FUNDC1, were significantly suppressed by sh-DNM1L treatment. Additionally, the expression of the antioxidant enzyme SOD1 was also notably down-regulated. In contrast, when glioma cells were subjected to OE-DNM1L treatment, these molecular expression changes exhibited an opposing trend, suggesting that DNM1L played a crucial role in modulating cellular autophagy and mitophagy (Fig. 10a - 10b; Supplementary Fig. 14a - 14b). Furthermore, the Mito-Keima adenovirus technology was employed to monitor the occurrence and progression of mitophagy by analyzing variations in fluorescence signals. Under neutral pH conditions, Mito-Keima emits green fluorescence upon excitation at 440 nm; conversely, under acidic pH conditions, it emits red fluorescence when excited at 561 nm. The alteration in the ratio of fluorescence signals (561 nm / 440 nm) served as a direct indicator of the dynamic changes occurring in mitophagy. The findings indicated that sh-DNM1L markedly diminished mitophagy levels, whereas OE-DNM1L significantly augmented mitophagy (Fig. 10c - 10d, Supplementary Fig. 14c - 14d). Similarly, sh-FIS1 exhibited pronounced inhibitory effects on both mitophagy levels and SOD1 expression in glioma cells; conversely, OE-FIS1 elicited comparable positive regulatory effects as OE-DNM1L. This suggested a potential synergistic role of FIS1 with DNM1L in modulating mitophagy (Fig. 10e - 10h, Supplementary Fig. 14e - 14h). Interestingly, OE-MFF significantly down-regulated the expression of ATG5, LC3-II, and various mitophagy-related markers (including PINK1, PARKIN, NIX, and FUNDC1), which was accompanied by an accumulation of p62 and a reduction in SOD1 expression in U87MG. This indicated that both mitophagy flux and activity were markedly inhibited. In comparison to OE-DNM1L and OE-FIS1, the impact of OE-MFF on mitophagy levels and SOD1 expression in glioma cells exhibited notable differences, further underscoring the distinct roles played by different molecules in modulating mitophagy and cellular oxidative states within glioma cells (Fig. 10i - 10l; Supplementary Fig. 14i - 14l). Such distinctions enhanced our comprehension of the intricate balance between mitochondrial dynamics and autophagy regulatory mechanisms, also motivated us to delve deeper into the complex network of interactions among these molecules and their collective influence on the biological behavior of glioma cells. These findings held significant implications for elucidating the pathogenesis of glioma as well as identifying novel therapeutic targets. Subsequently, we validated the regulatory functions of DNM1L/DRP1, FIS1, and MFF in autophagic flux and mitophagy within GSCs by conducting analogous treatment experiments, yielding results consistent with those observed in glioma cell lines (Supplementary Fig. 15a - 15l). This finding reinforced our previous observations in glioma cells and elucidated the roles of these molecules within GSCs, a subpopulation exhibiting characteristics akin to tumor stem cells.”

### 2. The authors revised the wording used to describe the experimental results, as suggested. However, the conclusions reported in paragraph 4 sound like an overstatement (lines 718-719). Please revise.

**For the in vivo experiments, for a more precise description of the methods, the authors should include the details on GSC xenograft experiments in materials and methods section.**

**[Response]**

1) In response to your concern regarding the potential exaggeration of the conclusions presented in Section 4 of our manuscript, we sincerely appreciate your feedback. We have resolved to revise this section. Our revised conclusions are as follows.

**Please check the lines 761 - 766.**

“These findings indicated that mitochondrial fission may be intricately linked to the biological behavior of gliomas, suggesting that modulation of mitochondrial dynamics could serve as a promising strategy for influencing glioma progression. Nevertheless, to elucidate the specific role and underlying mechanisms of mitochondrial fission in glioma advancement, further comprehensive research and validation are imperative.”

2) In response to your insightful feedback regarding the insufficient detail in the 'Materials and Methods' section of the GSCs xenograft experiment, we have meticulously refined the methodological content of the GSCs xenograft experiment, as elaborated below.

**Please check the lines 577 - 606.**

“We selected healthy male NOD/ShiLtJGpt-*Prkdc*^em26Cd52^*Il2rg*^em26Cd22^/Gpt (NCG) mice, aged between 4 - 6 weeks, utilizing six mice per group and establishing multiple experimental groups (including DNM1L-OE, DNM1L-KD, FIS1-OE, FIS1-KD, MFF-OE, MFF-KD, OE-NC, KD-NC) to comprehensively investigate the effects of various mitochondrial dynamics-related genes on glioma growth. Initially, we employed lentiviral vectors for stable transfection of target genes into two cell lines: the human glioma cell line U87MG—serving as a representative traditional glioma model—and GSCs, which provided a more cellular model closely resembling tumor origin and heterogeneity. We identified cell lines stably expressing the target genes through puromycin selection. Subsequently, we suspended the selected U87MG and GSCs at a concentration of 5 × 10^8^ cells/mL and injected 100 μL of this suspension (approximately 5 × 10^7^ cells) subcutaneously on the right dorsal side of each mouse. The condition of the nude mice was continuously monitored, and the subcutaneous tumor volume was measured on the 8th day when it reached a size between 50 - 100 mm^3^. Tumor length (L) and width (W) were measured every three days to calculate tumor volume (V = 0.5 × L × W × W). The experimental protocol was approved by the Laboratory Animal Welfare and Ethics Committee of Air Force Medical University (animal experiment ethics approval number: IACUC-20231262). Throughout the course of the experiment, we adhered rigorously to the guidelines established by the Laboratory Animal Welfare and Ethics Committee of the Air Force Medical University to ensure optimal welfare for the mice while monitoring tumor growth. We maintained that tumor weight did not exceed 10% of each mouse's body weight, with an average tumor diameter limited to no more than 16 mm, and ensured that tumor volume remained below 1500 mm³. Should any experimental mouse experience a weight loss exceeding 20% or develop ulcers resulting in infection or necrosis, the experiment would be terminated immediately, necessitating euthanasia of the animal. Ultimately, on completion day of the experiment, mice were euthanized via inhalation of 40% carbon dioxide, after which tumor tissues were promptly excised for subsequent analyses including molecular biology and histology.”

### 3. No further concerns.

### 4. The authors included in the main text the efficiency of lentiviral transfection on the modulation of the specific genes. The authors should better clarify if the reported percentages represent the fold change of the expression compared to its original level or compared to the corresponding NTC cells.

**[Response]**

In response to your inquiry regarding the specific interpretation of the percentage reported for gene regulation efficiency via lentiviral transduction, we recognize the necessity for a more precise and explicit elucidation. We would like to clarify that the percentages mentioned in the text pertain to changes in target gene expression levels relative to their baseline state, which is defined as the expression level observed in the corresponding NTC cells. To mitigate any potential confusion, we have revised this section of the text and explicitly indicated the reference point for these percentage changes.

For instance, the manuscript elucidates the efficacy of sh-DNM1L lentiviral transfection in down-regulating DNM1L as detailed below.

**Please check the lines 734 - 741.**

“The Western blot analysis revealed a significant reduction in the expression level of the target protein in cells transfected with specific shRNA lentivirus (sh-DNM1L) targeting the DNM1L gene, compared to those transfected with a non-specific control lentivirus (sh-DNM1L-NC). Specifically, the average expression level of the control group was normalized to 1, while that of the target protein in the sh-DNM1L group decreased to 0.274, indicating an approximate reduction of 72.6% relative to the control group.”

The regulatory efficiency reports pertaining to other genes align with those observed for sh-DNM1L.

**Please check the lines 741 - 745; the lines 792 - 796.**

We appreciate your meticulous review and insightful suggestions, which have significantly contributed to the enhancement of both the accuracy and clarity of our manuscript.

**Minor comments:**

### 1. The authors should include citation of Supplementary Table 1 in paragraph 2.

**[Response]**

We sincerely appreciate the comprehensive guidance offered by the reviewer concerning the recommendation to reference Supplementary Table 1 in the second paragraph. We have revised this section to explicitly acknowledge and cite the data or information contained in Supplementary Table 1. Specifically.

**Please check the lines 656 - 660.**

“In order to explore the underlying factors contributing to mitochondrial fragmentation and remodeling of MRC in HGG, we then performed bioinformatics analysis on RNA-seq data obtained from LGG, GBM (which was the highest type of HGG), and normal brain tissues using TCGA and GTEx databases (Specific information was provided in Supplementary Table 1)”。
